# Event-related potentials of automatic imitation are modulated by ethnicity during stimulus processing, but not during motor execution

**DOI:** 10.1038/s41598-018-30926-4

**Published:** 2018-08-24

**Authors:** Birgit Rauchbauer, Daniela M. Pfabigan, Claus Lamm

**Affiliations:** 1 Social, Cognitive and Affective Neuroscience Unit, Department of Basic Psychological Research and Research Methods, Faculty of Psychology, University of Vienna, Liebiggasse 5, 1010 Vienna, Austria; 2 Cognitive Science Research Platform, University of Vienna, Universitätsstraße 7, 1010 Vienna, Austria; 3 Laboratoire de Neurosciences Cognitive - UMR 7291, Aix-Marseille Université, CNRS, 3 Place Victor Hugo, Marseille, 13331 France; 4 Institut de Neurosciences de la Timone - UMR 7289, Aix-Marseille Université, CNRS, 27 Boulevard Jean Moulin, Marseille, 13005 France; 5 School of Psychological and Cognitive Sciences, PKU-IDG/McGovern Institute for Brain Research, Beijing Key Laboratory of Behavior and Mental Health, Peking University, 52 Haidian Road, 100871 Beijing, China

## Abstract

This study investigated neural processes underlying automatic imitation and its modulation by ethnically diverse hand stimuli (Black, White) using event-related brain potentials (ERPs). Automatic imitation relies on motor stimulus-response compatibility (SRC), i.e., response conflict caused by motoric (in)congruency between task-irrelevant hand stimuli and the required response. Our novel task aimed to separate two distinct neuro-cognitive processing stages of automatic imitation and its modulation by ethnicity: the stage of stimulus processing (i.e. perception), comprising presentation of stimulus ethnicity and SRC, and the stage of response execution (i.e. action). Effects of ethnicity were observed in ERPs of different stages of stimulus processing - during presentation of ethnicity (LPP) and SRC (N190, P3). ERPs at response execution, Pre-Motion Positivity (PMP) and Reafferent Potential (RAP), were only sensitive to congruency. The N190 results may index visual self-other distinction, while the neural timecourse of P3 and PMP variation could reflect a dynamical decision process linking perception to action, with motor initiation reflected in the PMP component. The PMP might further index motor-related self-other distinction regardless of ethnicity. Importantly, overt motor execution was not influenced by ethnically diverse stimuli, which suggests generalizability of the automatic imitation effect across ethnicities.

## Introduction

Imitation plays an important role in various domains of everyday life, such as when we imitate others consciously to acquire new behaviors, or mimic them in an unconscious and automatic manner during social interaction. Imitation and mimicry potentially share the same basis of mechanisms, such as the direct link between the perception and execution of an action, which can be investigated experimentally via automatic imitation paradigms (^1^, but see^2^). Automatic imitation has been suggested to represent a laboratory model of mimicry^1^, repeatedly investigated with the imitation-inhibition task^3^. It refers to the observation that the perception of an action interferes with the execution of an instructed movement in stimulus-response compatibility (SRC) paradigms. As such, the overt behavioral response may be sped up or slowed down by the mere presentation of a task-irrelevant identical or conflicting movement, through a direct link between perception and execution of an action. Automatic imitation has been shown to be modulated by social context cues (see e.g.^3–11^), yet, presenting the nature of the task-irrelevant hand at the wrist only, such as in a study showing human versus robotic hands, has been found to not alter the automatic imitation effect^11^. It stands to reason that the same effect could be observed by adding a manipulation of ethnicity to the task-irrelevant hands (i.e. wrists), allowing to investigate generalizability of automatic imitation across ethnicities. Nevertheless, a uniform reaction time measure, even if not modulated by the presentation of humanness^11^ or potentially ethnicity (on the wrist), may obscure neural dynamics of underlying distinct neuro-cognitive processes involved in the expression of the overt behavioral response. Event-related brain potentials (ERPs) allow to highlight the influence of neural processes during the stage of perceptual stimulus processing, including the presentation of stimulus ethnicity and of SRC (i.e. perception) and directly at the stage of overt behavioral response execution (i.e. action). To this date the neural dynamics of automatic imitation have only recently started to be investigated (see for example^12^). The present study aims to extend these studies to the presentation of ethnically diverse hand stimuli to establish potential generalizability of the automatic imitation effect across ethnicities.

## Ethnic imitation-inhibition task (EIIT)

**Figure 1:**
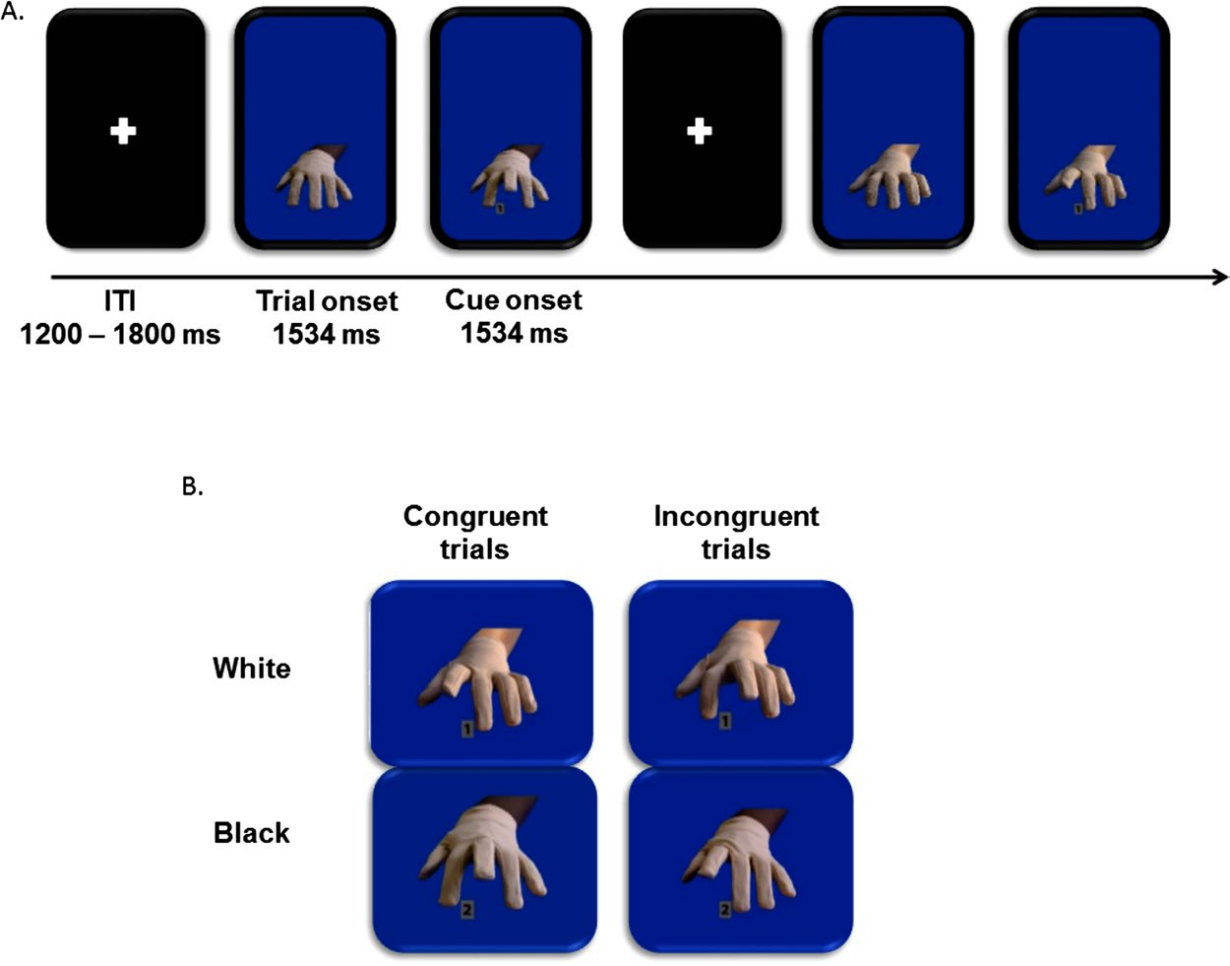
Experimental setup of EIIT. (**A**) Timeline of ethnic imitation-inhibition task (EIIT). (**B**) Ethnic (Black and White) hand stimuli in congruent and incongruent trials. Hand pictures by BR.

The present study used a modified version of the imitation-inhibition task by Brass *et al*.^3^ by adding ethnically diverse hand stimuli (i.e. the task-irrelevant stimuli) – the ethnic imitation-inhibition task (EIIT). First, the EIIT presented solely the White and Black task-irrelevant hand stimuli, without any task-relevant number cue. Ethnicity of the hand was visible only at the wrist since the hand was covered with a beige glove (for details see section 1.2 and Fig. 1). Second, the target cue was presented, indexing either an index or middle finger lifting movement. Concurrently to the presentation of the target cue, a finger-lifting movement of the hand on the screen was presented. The movement was either congruent or incongruent to the target cue and thus facilitated or slowed down actual response execution (see methods for details). This allowed us to investigate the neural dynamics during the stage of stimulus processing (i.e. first, perceptual processing of salient ethnicity/color cues and second, concurring task-irrelevant actions, thus creating the SRC effect) and directly at the stage of response execution giving rise to the automatic imitation effect. In addition, we explored whether the automatic imitation effect, as well as movement inhibition and facilitation (both baseline adjusted) were modulated by the presentation of ethnically diverse hands on a behavioral level.

We focused on the following ERP components potentially engaged in the various stages of task processing, i.e. perceptual stimulus ethnicity and SRC processing and response execution:

The stage of perceptual stimulus processing was divided into two parts: the presentation of stimulus ethnicity and SRC presentation. During ethnicity presentation we were interested in the N100 component potentially reflecting enhanced attentional focus^13^, and the late positive potential (LPP) indexing heightened motivational relevance of the presented stimuli. Both ERPs have been shown to differentiate between ethnicity of stimuli^14,15^. The N100 thereby shows more negative^15^, the LPP more positive amplitudes associated with other ethnicities than one’s own^14^. During SRC presentation, two ERPs, the N190 and the P3, were investigated. The N190, a component suggested to reflect visual categorization of human body parts^16^, has recently been documented as sensitive to interference in the imitation-inhibition task, with more negative left-hemispheric amplitudes for incongruent trials^12^. Specifically, it has been suggested to index low-level visual categorization and self-other discrimination processes^12^. The amplitude of the parietal P3 has been shown to be sensitive to the SRC mappings in Simon and joint action tasks, enhanced for congruent as compared to incongruent trials (the go-P3 in go/no-go tasks)^17–20^. It has been suggested that the P3 SRC difference reflects perceptual interference and the response selection conflict^18,19^. The result of enhanced P3 amplitudes on congruent trials has been replicated in imitation-inhibition tasks^12,21^, and interpreted as reflecting social-cognitive self-other discrimination processes^12^. Furthermore, it has also been suggested that the P3 component reflects information processing functions of the locus coeruleus – noradrenergic system, sensitive to motivational significance of events and facilitating behavioural responses^22^.

For the period of response execution, we investigated both pre- and post-movement ERPs. The pre-motion positivity (PMP)^23^, also referred to as P-50^24,25^, occurs immediately before movement onset^23^. It has been suggested to reflect motor commands for movement initiation^23,26,27^, yet may also index inhibition of an imitative movement^28^. To extend Deschrijver *et al*.’s^12^ findings, we were particularly interested in investigating the PMP and its role in movement initiation or inhibition in the automatic imitation task. The PMP can be observed at the end of the well-known motor preparation ERP readiness potential (RP^29^) as a positive amplitude shift with a rather ipsilateral topography for finger movements^23^. As post-movement ERP, we investigated the reafferent potential (RAP)^23^, also known as post-movement positive potential (PMPP)^30^ or P + 90^24,25^, peaking around 100 ms after movement onset at parietal and precentral areas. This component may reflect reafferent sensory information following movement production and is considered a “resolution potential”, clearing preceding and subsequent electro-cortical negativities^24,30^.

## Hypotheses

Since this was to our knowledge the first study investigating the ERPs of automatic imitation using ethnically diverse hand stimuli, the majority of the following hypotheses were exploratory: At the stage of perceptual stimulus processing, during presentation of stimulus ethnicity, we hypothesized to observe a distinction of White and Black stimuli, expressed in more negative N1 and more positive LPP amplitudes for Black stimuli^14,15^. During SRC presentation, we expected more negative left-hemispheric N190 amplitudes for incongruent trials^12^. We expected the presentation of Black hands to evoke enhanced visual categorization and low-level visual discrimination processes compared to White hand stimuli, due to more salient color of the presented Black hand stimuli. Thus, we expected enhanced negative N190 amplitudes for Black as compared to White hands, alongside a larger congruency effect (i.e. difference between incongruent and congruent trials) for Black (as compared to White) hands. Regarding the P3 component, we expected more positive amplitudes for congruent compared to incongruent trials^12,17–21^. The hypothesis regarding the directionality of P3 modulation by ethnicity was open: If the P3 component reflects enhanced perceptual interference and response selection we expected more positive amplitudes for White as compared to Black stimuli, as well as an interaction effect of ethnicity and congruency. Specifically, we expected the congruency effect to be larger for White as compared to Black hands. This should be due to the enhanced perceptual overlap in White hand trials (i.e. in-group hands; since all participants were White), and a resulting enhanced interference for response execution (i.e. facilitation on congruent, inhibition on incongruent trials). In this case, it could be suggested that the P3 reflects social-cognitive self-other distinction. On the other hand, if the P3 component is sensitive to motivational significance^22^, we expect enhanced P3 amplitudes for Black as compared to White hands. Black hands in our study constituted out-group hands and may, due to their salient and distinct color/ethnicity compared to one’s own hands, represent the motivationally more significant event, comprising facilitated stimulus evaluation. In this case, and in line with the N190 hypothesis, we expect a larger congruency effect for Black than White hand trials.

At the stage of response execution, we expected the PMP and the RAP components to differentiate between congruent and incongruent trials. The hypotheses for the PMP were also open: On the one hand, if the PMP in the automatic imitation task indexes movement inhibition in general (in line with^28^), we expect more positive PMP amplitudes on incongruent trials. On the other hand, if the PMP reflects movement initiation per se, which should be facilitated on congruent trials, this will be reflected in more positive PMP amplitudes on congruent trials. In this case, more positive PMP amplitudes are expected for congruent compared to incongruent trials. We expect the RAP to be highly linked to the PMP and to differentiate between congruency according to PMP amplitudes. Since the PMP and RAP reflect movement execution processes, we focus our hypotheses on the action-related congruency processing and do not have specific hypotheses regarding ethnicity or the interaction of ethnicity and congruency. Moreover, we explore whether ERPs at the stage of stimulus processing or response execution predict the automatic imitation effect and the corresponding RAP component. Furthermore, in an exploratory approach, we aimed to investigate whether ERP component latencies and/or amplitudes predicted the individual mean RT’s on congruent and incongruent trials per ethnicity, as P3 latencies have been associated with RTs (e.g.^31,32^). Fourth, in line with Press *et al*.^11^ we expected no modulation of the automatic imitation effect, as well as baseline adjusted movement inhibition (II_c_) and facilitation (FI_c_) indices due to ethnicity.

## Results

### Behavioral Results

#### Attitudes towards Black Scale

Participants rated their attitude towards Blacks in the middle of the scale (M = 4.09, SE = 0.39). No outliers were observed and thus data of all 29 participants was available for further analysis.

#### EIIT

The dependent t-test comparing the automatic imitation effect (i.e. mean reaction time differences incongruent minus congruent) for the factor Ethnicity showed no significant difference (t(28) = −0.116, p = 0.910). The two-way repeated measurement ANOVA of mean RTs showed a significant main effect of Congruency (F(2, 56) = 86.29, p < 0.001, partial η2 = 0.85). In line with previous results (e.g.^3^), mean RTs on incongruent trials were longer than on congruent (t(28) = 11.09, p < 0.001; incongruent: M = 543.59 ms, SE = 12.02 ms, congruent: M = 480.06 ms, SE = 10.60 ms), and baseline trials (t(28) = 5.16, p < 0.001; M = 517.17 ms, SE = 10.76 ms). Also, in line with previous studies (e.g.^3^) mean RTs on congruent trials were faster than on baseline trials (t(28) = 10.79, p < 0.001). Further we found a trend effect of Ethnicity with longer reaction times for Black than White hand stimuli (F(1, 28) = 3.18, p = 0.085, partial η2 = 0.102; Black: M = 514.99, SE = 10.769; White: M = 512.22, SE = 10.51) in the absence of an interaction effect (F(2, 56) = 1.554, p = 0.220).

We also carried out dependent t-tests comparing the FI_c_ and II_c_ per Ethnicity. This did not reveal significant results (all t-values (28) ≤ 1.651, all p-values ≥ 0.110).

## EEG Results

### Perception: Stimulus processing

#### Ethnicity presentation (stimulus-locked frame 1)

N100: The dependent t-test comparing N100 amplitudes in response to Ethnicity showed no significant effect (t(28) = 0.839, p = 0.409).

**Figure 2:**
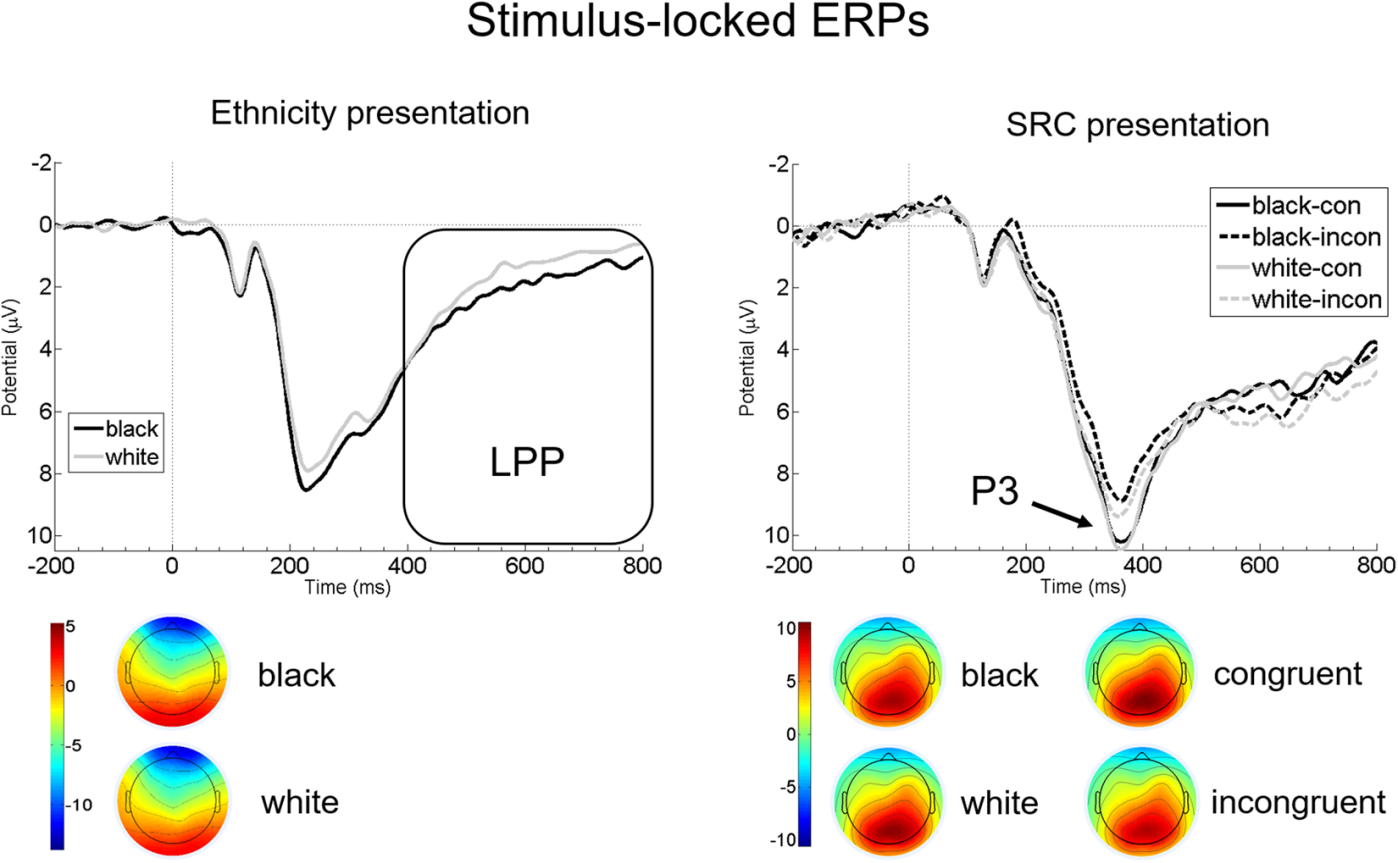
Grand average of stimulus-locked ERPs (LPP and P3). Left panel: Mean LPP amplitudes 400–800 ms after onset of Frame 1 (rectangle) at merged electrodes R24, R30, L24, L28 and Cz (L30) per Ethnicity (White vs. Black). Right panel: P3 amplitudes after onset of Frame 2 (arrow) at merged electrodes R25, R26, R29, R30, L22, Pz (L26), L27, L28, and Cz (L30) per Congruency (congruent [con] vs. incongruent [incon]) and Ethnicity (White vs. Black). Negative amplitudes are drawn upwards by convention. Stimulus presentation started at 0, indicated by a ticked vertical line. For demonstrational purposes, only the first 800 ms of the frames are depicted. Below, scalp topographies of mean LPP (left panel; 400–800 ms after Frame 1 onset) and P3 amplitudes (right panel; 300–400 ms after Frame 2 onset) are presented separately for significant main effects.

LPP: The dependent t-test comparing LPP amplitudes per Ethnicity revealed significant differences (t(28) = 4.467, p < 0.001, Cohen’s *d* = 0.362). LPP mean amplitudes were more positive for Black compared to White ethnic stimuli (Black: M = 2.367, SE = 0.262, White: M = 1.763, SE = 0.35) (see Fig. 2).

#### SRC presentation (stimulus-locked frame 2)

**Figure 3:**
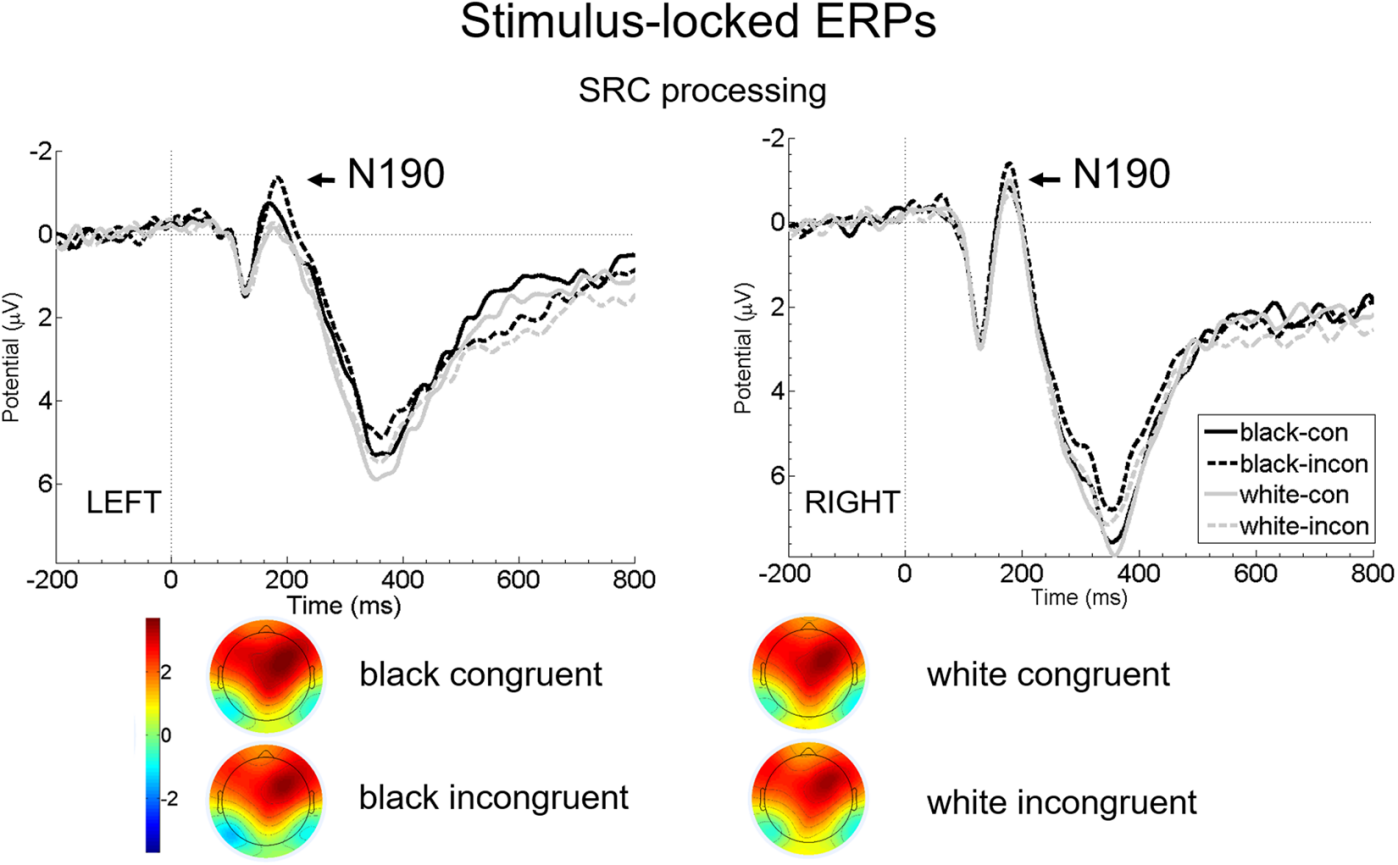
Grand average of stimulus-locked N190. Left panel depicts left hemispheric, right panel depicts right hemispheric N190 amplitudes after onset of Frame 2 (arrow) at merged electrodes R22, R27, R30 (right hemisphere) and L20, L24, L28 (left hemisphere) per Ethnicity (White vs. Black) and Congruency (congruent [con] vs. incongruent [incon]). Negative amplitudes are drawn upwards by convention. Stimulus presentation started at 0, indicated by a ticked vertical line. For demonstrational purposes, only the first 800 ms of the frames are depicted. Below, scalp topographies of N190 per Ethnicity (White, Black) and Congruency (congruent, incongruent) are presented merged over both hemispheres.

N190 peak amplitudes: The 2 × 2 × 2 repeated measures ANOVA with the factors Ethnicity, Congruency, and Hemisphere revealed a significant result for the factor Ethnicity, with more negative amplitudes for Black than White hands (F(1, 28) = 9.931, p = 0.004, partial η2 = 0.262; White: M = −1.478, SE = 0.334; Black: M = −1.977, SE = 0.389). Furthermore we found a trend result for the interaction effect of Ethnicity x Hemisphere (F(1, 28) = 3.040, p = 0.092, partial η2 = 0.098). Bonferroni corrected planned pairwise comparisons revealed no significant difference in N190 peak amplitudes between corresponding conditions per hemisphere (all p-values ≥ 0.498). The main effect of Congruency was not significant (F(1, 28) = 2.185, p = 0.151, partial η2 = 0.072), as were the interaction effects Ethnicity x Congruency (F(1, 28) = 0.217, p = 0.645, partial η2 = 0.008), Hemisphere x Congruency (F(1, 28) = 0.219, p = 0.643, partial η2 = 0.008) and the three-way interaction of Ethnicity x Congruency x Hemisphere (F(1, 28) = 0.438, p = 0.514, partial η2 = 0.015) (see Fig. 3).

P3 peak amplitudes: The 2 × 2 repeated measures ANOVA with the factors Ethnicity and Congruency showed a significant main effect of Congruency with more positive P3 amplitudes for congruent than incongruent stimuli (F(1, 28) = 27.159, p < 0.001, partial η2 = 0.492; Congruent: M = 11.744, SE = 0.753; Incongruent: M = 10.63, SE = 0.727). The main effect of Ethnicity was not significant (F(1, 28) = 0.076, p = 0.785, partial η2 = 0.003), but a significant interaction for the factors Ethnicity and Congruency was observed (F(1, 28) = 7.465, p = 0.011, partial η2 = 0.21). Bonferroni corrected planned pairwise comparisons revealed a significant difference between congruent and incongruent trials for Black hands, with more positive amplitudes for congruent than incongruent trials (t(28) = 5.372, p < 0.001; Congruent Black: M = 11.986, SE = 0.789; Incongruent Black: M = 10.323, SE = 0.748). The congruency difference for White hands, with larger P3 peak amplitudes for congruent than incongruent trials did not reach significance after Bonferroni correction (t(28) = 2.051, p = 0.05; Congruent White: M = 11.504, SE = 0.729; Incongruent White: M = 10.936, SE = 0.729). Congruent trials were comparable across ethnicity, with no significant difference found between White and Black hands (t(28) = 1.389, p = 0.176;). Furthermore, results showed (by trend) more positive peak amplitudes for incongruent White than Black trials (t(28) = 2.322, p = 0.028). (see Fig. 2).

### Action: Response execution (response-locked frame 2)

**Figure 4:**
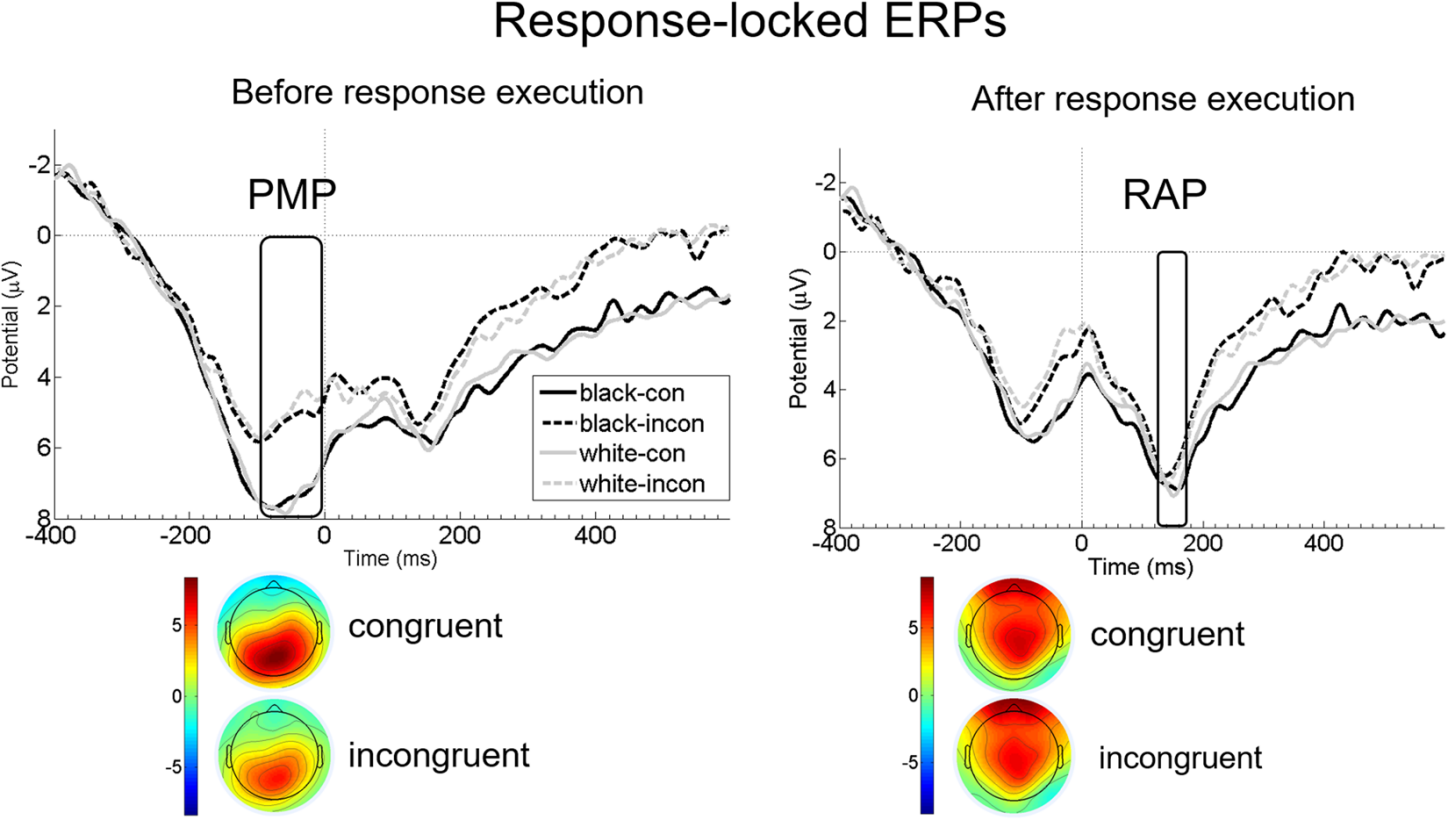
Grand average of response-locked ERPs (PMP and RAP). Left panel: Mean PMP amplitudes 100 ms prior until the button press (rectangle) at merged electrodes R19, CPz (R24), R25, R29, L22, Pz (L26), and L27, per Congruency (congruent [con]vs. incongruent [incon]) and Ethnicity (White vs. Black). Right panel: Mean RAP amplitudes 126–176 ms after response execution (rectangle) at merged electrodes CFz (R14), Cz (L16), CPz (R24), R15, R16, R19, R25, as well as L12 and L17 per Congruency (congruent [con] vs. incongruent [incon]) and Ethnicity (White vs. Black). Negative amplitudes are drawn upwards by convention. Response execution started at 0, indicated by a ticked vertical line. Below, scalp topographies of mean PMP (left panel; −100 ms prior until button press) and RAP amplitudes (right panel; 126–176 ms after response execution) are presented separately for significant main effects.

Pre-motor Positivity (PMP): The 2 × 2 repeated measures ANOVA with the factors Ethnicity and Congruency revealed a significant main effect of Congruency, showing more positive PMP amplitudes for congruent than incongruent trials (F(1, 28) = 27.97, p < 0.001, partial η2 = 0.50; Congruent: M = 7.37, SE = 0.73; Incongruent: M = 5.13, SE = 0.70) (see Fig. 4). No other effects were significant (all p-values ≥ 0.37) (see Fig. 4).

Reafferent Potential (RAP): The 2 × 2 repeated measures ANOVA with the factors Ethnicity and Congruency revealed a significant main effect for the factor Congruency. More positive RAP amplitudes were elicited during congruent than incongruent trials (F(1, 28) = 5.508, p = 0.03, partial η2 = 0.16; Congruent: M = 5.104 SE = 0.79; Incongruent: M = 4.14, SE = 0.73) (see Fig. 4). No other effects were significant (all p-values ≥ 0.27) (see Fig. 4).

### Correlation analyses

Correlation analyses were carried out between corresponding condition specific ERP mean and peak amplitudes showing a significant effect of Congruency and the respective behavioral response. P3 peak amplitudes (all p-values ≥ 0.131) per ethnicity condition were not significantly correlated with corresponding facilitation (FIc) or inhibition (IIc) indices. Congruent PMP amplitudes and the facilitation indices (FIc) were not correlated per ethnicity (all p-values ≥ 0.253). The inhibition indices (IIc) per ethnicity condition were negatively correlated with incongruent PMP amplitudes for Black and, by trend, for White hands (Black: r = − 0.554, p = 0.002; White: r = −0.335, p = 0.075). Congruent RAP amplitudes of both ethnicities were not correlated with the corresponding facilitation indices (FIc), as were incongruent RAP amplitudes of White hands with the respective inhibition index (IIc) (all p-values ≥ 0.142). In contrast, for Black hands, incongruent RAP amplitudes and the inhibition index (IIc) were significantly correlated (r = −0.53, p = 0.003).

### Multiple Regression Models (on RT and ERP difference measure separated per Ethnicity)

For White hand stimuli, results revealed, first, PMP mean amplitude differences of incongruent minus congruent trials as the best predictor of the magnitude of the individual automatic imitation effect (see Table 1 details) (Regression analyses were performed using difference measures of mean amplitudes in incongruent minus congruent trials per ethnicity to mirror the automatic imitation effect for all models, respectively mean amplitudes of the LPP per ethnicity). For Black hand stimuli, the congruency difference measures of the N190 peak amplitudes alongside the PMP (both for Black trials) best predicted the individual automatic imitation effect on presentation of Black hand stimuli (see Table 2 for details). Second, PMP congruency difference measure amplitudes also predicted RAP congruency difference measure amplitudes on the presentation of both White and Black hand stimuli (see Tables 3 and 4 for details) (Difference measure of ERP components were obtained by subtracting mean amplitudes of incongruent minus congruent trials, to reflect the behavioral automatic imitation effect. See methods for details).

**Table 1:** Multiple regression (stepwise forward) for the automatic imitation effect for the presentation of White hands; Independent Variables: mean amplitudes of LPP, Difference measures (incongruent minus congruent trials) of N190, P3 and PMP per presentation of White hands.

	B	SE B	β
Modell 1			
Constant	53.681	6.763	
PMP (difference wave)	−3.962	1.929	−0.368*

Note: R^2^ = 0.135 for Modell 1; *p = 0.05.

**Table 2:** Multiple regression (stepwise forward) for the automatic imitation effect for the presentation of Black hands; Independent Variables: mean amplitudes of LPP, Difference measures (incongruent minus congruent trials) of N190, P3 and PMP per presentation of Black hands.

	B	SE B	β
Modell 1			
Constant	46.974	7.907	
PMP (difference wave)	−8.153	2.494	−0.532*
Modell 2			
Constant	55.933	8.277	
PMP (difference wave)	−7.114	2.355	−0.465*
N190 (difference wave)	16.472	7.072	0.358**

Note: R^2^ = 0.532 for Modell 1; *p = 0.003; ΔR^2^ = 0.638 for Modell 2 (p < 0.001); *p = 0.006, **p = 0.028.

**Table 3:** Multiple regression (stepwise forward) for Reafferent Potential (RAP) for the presentation of White hands; Independent Variables: mean amplitudes of LPP, Difference measures (incongruent minus congruent trials) of N190, P3 and PMP per presentation of White hands.

	B	SE B	β
Modell 1			
Constant	0.670	0.420	
PMP (difference wave)	0.741	0.120	0.766**

Note: R^2^ = 0.586 for Modell 1; **p < 0.001.

**Table 4:** Multiple regression (stepwise forward) for Reafferent Potential (RAP) for the presentation of Black hands; Independent Variables: mean amplitudes of LPP, Difference measures (incongruent minus congruent trials) of N190, P3 and PMP per presentation of Black hands.

	B	SE B	β
Modell 1			
Constant	0.809	0.429	
PMP (difference wave)	0.781	0.135	0.743**

Note: R^2^ = 0.552 for Modell 1, **p < 0.001.

### Multiple Regression Models (per condition)

For congruent trials, P3 peak amplitude latencies together with the mean amplitude for the PMP (per condition) predicted individual RTs on both Black and White trials (see Tables 5 and 6). For incongruent trials, the PMP mean amplitude alone predicted the individual RTs for the corresponding Black and White trials (see Tables 7 and 8). (Regression analyses were performed using z-transformed, standardized data in order to compare the influence of P3 peak latencies (i.e; temporal scale; ms) and peak and mean ERP amplitudes (i.e. µV) on (also standardized) RTs (i.e. ms) per condition (see methods for details).

**Table 5:** Multiple regression individual mean reaction time on Black congruent trials Independent Variables: mean amplitude of LPP, peak amplitude of N190 and P3, P3 peak latency and PMP mean amplitude for Black congruent trials (all data normalized z-scores).

	B	SE B	β
Modell 1			
Constant	−1.081E-15	0.160	
P3 latency	0.112	0.230	0.384*
PMP mean amplitude	−0.515	0.211	−2.439**

Note: R^2^ = 0.626 for Modell 1, *p = 0.032, **p = 0.23.

**Table 6:** Multiple regression individual mean reaction time on White congruent trials. Independent Variables: mean amplitude of LPP, peak amplitude of N190 and P3, P3 peak latency and PMP mean amplitude for White congruent trials (all data normalized z-scores).

	B	SE B	β
Modell 1			
Constant	3.154E-15	0.151	
P3 latency	0.620	0.183	0.620**
PMP mean amplitude	−0.536	0.237	−0.536*

Note: R^2^ = 0.691 for Modell 1, **p = 0.003, *p = 0.034.

**Table 7:** Multiple regression individual mean reaction time on Black incongruent trials. Independent Variables: mean amplitude of LPP, peak amplitude of N190 and P3, P3 peak latency and PMP mean amplitude for Black incongruent trials (all data normalized z-scores).

	B	SE B	β
Modell 1			
Constant	3.564E-15	0.158	
PMP mean amplitude	−0.638	0.190	−0.638**

Note: R^2^ = 0.659 for Modell 1, **p = 0.003.

**Table 8:** Multiple regression individual mean reaction time on White incongruent trials Independent Variables: mean amplitude of LPP, peak amplitude of N190 and P3, P3 peak latency and PMP mean amplitude for White incongruent trials (all data normalized z-scores).

	B	SE B	β
Modell 1			
Constant	9.645E-16	0.158	
PMP mean amplitude	−0.521	0.201	−0.521*

Note: R^2^ = 0.655 for Modell 1, *p = 0.017.

## Discussion

The main objective of the present study was to investigate the dynamics of the neuro-cognitive processes associated with automatic imitation and their modulation by ethnically diverse hand stimuli. In addition to behavioral measures of automatic imitation, we analysed ERP measures during the stages of stimulus processing and response execution. This also allowed to determine whether the individual magnitude of the automatic imitation effect, the individual RTs on congruent and incongruent trials per ethnicity, as well as the RAP component, would be predicted by ERPs related to stimulus processing or actual response execution. In terms of behavioral results, we observed, as expected, faster RT on congruent, and slower RT on baseline and incongruent trials. However, the automatic imitation effect, as well as the baseline adjusted facilitation and inhibition indices, were not modulated by ethnicity. In terms of neural dynamics, we observed effects of ethnicity early during the first stage of perceptual stimulus processing (i.e. the presentation of an ethnically diverse hand), alongside SRC effects later during this period. Specifically, we found more pronounced LPP mean amplitudes during ethnicity presentation, and higher N190 peak amplitudes during congruency presentation for Black hand trials. N190 peak amplitudes did not show an effect of congruency or the expected interaction effect between ethnicity and congruency. P3 peak amplitudes differentiated significantly between congruent and incongruent Black, but not White hand trials.

During the stage of response execution, only congruency was reflected in pre- and post-response ERPs, with more positive amplitudes both before (PMP) and after response execution (RAP) on congruent trials. The inhibition index was significantly negatively associated with PMP amplitudes for Black and by trend for White hands, and with RAP amplitudes for Black stimuli. Only PMP mean amplitude difference measures predicted the behavioral automatic imitation effect reliably for the presentation of White hands. The model combining amplitude difference measures of the N190 and PMP was the best predictor for the automatic imitation effect for the presentation of Black hands. PMP mean amplitude difference measures best predicted the amplitude of the RAP mean amplitude difference measure for both the presentation of White and Black hands. Individual’s mean RTs on congruent trials per ethnicity were best predicted by the P3 peak latencies and the PMP mean amplitudes (standardized data). Individual’s mean RTs on incongruent trials per ethnicity were best predicted by the PMP mean amplitudes alone. In the remainder of this paper, we will interpret and discuss these findings.

### Perception: Stimulus processing of ethnicity & SRC

During the stage of stimulus processing our results revealed ethnicity-specific differences, which we will lay out in detail for both the presentation of ethnicity and SRC. For the period of ethnicity presentation without SRC information, enhanced amplitudes were found for the LPP, which has been shown sensitive to biologically relevant information, such as the presentation of threatening information^33–36^, and also been associated with categorization processes of ethnic stimuli^14^. According to findings of implicit associations with Blacks and threat in implicit association tests (IATs) (see for example^7,8,37–40^), it could be argued that LPP amplitude variation may reflect the processing of threatening stimuli. Yet, since in the present study only pictures of Black hands were presented (and not e.g. threatening facial expressions or body postures), we do not find it very plausible that these stimuli were associated with threat by the participants. Rather, the differentiation of LPP amplitudes in our study suggests categorization processes of stimulus ethnicity, which might be due to the stimulus salience, color, and/or relevance during the stage of perceptual stimulus processing. In contrast, N100 amplitudes were comparable for Black and White hand stimuli. Thus, the current manipulation might have been too subtle to be reflected in very early attention deployment (14).

Also during SRC presentation, our results revealed enhanced negative peak amplitudes for Black hand stimuli in the N190 component. Furthermore, regression analysis showed that the difference measure of the N190 predicted the behavioral automatic imitation effect only for Black hand trials. Yet, the present results could not replicate the previously reported result of enhanced N190 amplitudes for incongruent compared to congruent trials^12^. It has been suggested that the N190 reflects the extraction of information relevant for categorization of the human body^16^. Thus, Black hands may have evoked enhanced categorization processes in our exclusively White-Caucasian participants. In line with this, the extrastriate body area (EBA), the potential generator of the N190^16^, has been shown activated during low-level visual self-other discrimination^41^. The N190 in the present study may index low-level visual self-other distinction on trials presenting Black hands, regardless of SRC processing. The present study thus extends Deschrijver *et al*.s’^12^ finding of a N190 congruency effect in an imitation-inhibition task to the color/ethnicity of the displayed task-irrelevant hands. In the case of the EIIT, the color/ethnicity of the task-irrelevant hand could have constituted the more salient processing feature to index visual self-other distinction, than SRC, which was the defining feature for the N190 differences in Deschrijvers study. Considering the visual saliency of Black hands, we argue that it is not visual processing effort giving rise to the enhanced N190 amplitude, as they should be easily processed due to their perceptual and attentional saliency. Rather we suggest that the N190 indexes processes of visual self-other distinction. Nevertheless, it could be interesting for future studies to collect data of Black participants with this task, to investigate whether for Black participants, White task-irrelevant hands could constitute the more salient processing feature, thus adding a social component to this low-level visual self-other distinction component. Furthermore, in Black hand trials, the N190 predicted the automatic imitation effect alongside the PMP. This suggests the involvement of visual self-other distinction influencing the individual automatic imitation effect alongside the PMP, which, as we will discuss later on, may, in our task, index motor-related self-other distinction. Taken together, we suggest that low-level visual self-other distinction, triggered by visual categorization of differently colored/ethnic hands, evoked the observed N190 color/ethnicity differences, regardless of SRC at this stage of stimulus processing.

Furthermore, during the stage of stimulus processing, specifically during SRC presentation, we found a larger congruency effect in P3 peak amplitudes for Black hand trials. Our results thus extend previous findings of more pronounced P3 amplitudes for congruent compared to incongruent trials in Simon^18,19^, joint action^17,20^ and imitation-inhibition tasks^12^ to the ethnicity of the presented hands. The congruency effect reflected in the P3 amplitude was enhanced for Black hand trials. This suggests that the P3 may encode motivationally significant events, which, in the present study, were the saliently colored/ethnic Black hands during SRC processing, potentially contributing to a dynamic decision process of linking perception to action^31,42–45^ (see further discussion below). It could be of interest to investigate whether this effect is based on a purely perceptual effect of salient color or is based on a social categorization of in- and out-group hands - the presentation of and required adaptation of behavior to out-group hands constituting the motivationally more important event. Thus, including both White and Black participants in a study using the EIIT, could show whether this effect would be reversed in Black participants presented with White task-irrelevant hands. This would suggest that the motivational salience is driven by an in- and out-group effect and not just by the salience of the colored wrist. Furthermore this could be extended using hands of same color/ethnicity and modulating group membership with a minimal group paradigm^46^, indexing in- and our-group belonging with differently colored wrist bands. This would clarify the role of the stimulus-locked P3 component in indexing motivationally salient events in the automatic imitation task based on social group categories or purely perceptual effects. Of note, Deschrijver *et al*.^12^ suggested that the P3 may reflect self-other distinction. However, in the context of our experimental paradigm, we suggest that the P3 may not reflect social-cognitive self-other distinction processes here, as this should have been reflected in a larger congruency effect for White hands, indexing enhanced efforts to discriminate one’s own from a movement of a hand of the same ethnicity. Rather, as we will discuss further below, we suggest that the PMP might reflect purely motor-related self-other distinction. It is suggested that in our study, by adding contextual information in the form of differentially colored/ethnical wrists, the process for self-other distinction occurred later in time than proposed by Deschrijver *et al*.^12^. This could specifically be the case if we consider a potential overlap of the PMP with a response-locked P3, which we will discuss below.

Taken together, the early stages of perceptual stimulus processing, specifically at the time of presentation of ethnicity (LPP) revealed categorization processes that were specifically involved in coding salient color/ethnicity features of the stimuli at hand. During early SRC processing, visual self-other distinction processes due to color/ethnicity of the presented hand were indexed by the N190. At a later processing window during this stage (P3), motivationally salient information was encoded, revealing an enhanced congruency effect for Black hand trials. Furthermore, as discussed in detail below, the stimulus-related P3 may, together with the PMP, also be seen as part of a dynamically evolving decision process linking the perception of motivationally salient information and motor preparation.

### Action: The stage of response execution

During the stage of response execution, both PMP and RAP components differentiated between movement congruency only, without effects of ethnicity. We found enhanced positive-going deflections of both components for congruent trials. In addition, incongruent PMP amplitudes were correlated negatively with the inhibition index and thus movement inhibition for both Black and, by trend, White stimuli. It has been suggested that the PMP reflects the motor command to initiate movement^26,27^. Considering the present results of enhanced amplitudes on congruent trials and a negative correlation with movement inhibition, it can be suggested that the PMP indexes the motor command for movement initiation in the EIIT in general. Moreover, the PMP was a reliable predictor of the RAP, which could furthermore underline its role as exerting motor commands for movement initiation. The PMP also predicted the automatic imitation effect for both the presentation of White, as well as, alongside the N190, for Black hand stimuli. Thus, for the presentation of White hands, the present results suggest that automatic imitation is directly mediated at the stage of movement initiation. In the case of the presentation of Black stimuli it is suggested that the overt behavioral automatic imitation response is influenced by neural processes both at the stage of stimulus processing, reflected in the N190, and response execution. More specifically, the automatic imitation response on Black hand trials may be mediated by visual categorization processes (N190) at the stage of SRC presentation and directly at the stage of movement initiation (PMP).

Furthermore, PMP amplitudes best predicted individual mean RTs, together with the P3 peak latency on congruent, and alone on incongruent trials, for both ethnicities. Extending Deschrijver *et al*.’s notion we suggest that rather than the stimulus-locked P3, the PMP to index purely motor-related self-other distinction without interference of ethnicity in the present task. As such, this could place motor-related self-other distinction at the end of a dynamic neural process linking perception to action. As mentioned above, in the case of the present study, by adding contextual information on the color/ethnicity of the presented wrists, self-other distinction might take place later on in this dynamic decision process. Future studies should aim at disentangling the occurrence of motor-related self-other distinction and its potential modulation through contextual variables to shed light on the dynamical decision process linking perception to action. Of note, recent literature suggests to view the P3 component not only from a stimulus-locked, but also from a response-locked perspective (see e.g.^31,42–45^). In the current study, we suggest that the assessed PMP component could partly overlap with stimulus- and response-locked portions of the P3 component on a level of dynamical processing, considering also the shared topography of the PMP and the P3.

RAP amplitudes showed enhanced positive amplitudes for congruent trials, yet RAP amplitudes correlated negatively with movement inhibition (i.e. the II_c_ index) for Black stimuli. It has been suggested that the RAP represents reafferent sensory information following movement production^24^, potentially generated in the motor cortex^30^. The present results thus suggest that the RAP component reflects sensory feedback about congruency with the imitated hand, yet, in the case of distinct color/ethnicity of the presented hand, specifically related to the inhibition of imitative movements. The prediction of the RAP by the PMP suggests that the processes of movement initiation (PMP) is directly associated with the individual reafferent sensory feedback of the executed movement, reflected in the RAP.

Using White and Black hand stimuli in beige gloves, our behavioral results, by trend, revealed that individual RTs were influenced by the color/ethnicity of the presented hand, while the automatic imitation effect, as well as facilitation and inhibition indices were not modulated by the ethnicity modulation. This is in line with a study investigating automatic imitation of human versus robotic hands, also showing the nature of the hand only at the wrist^11^. Similar to our study, manipulating the wrists of humans and robots did not modulate the behavioral response.

### Linking perception to action: P3 & PMP

It has been suggested that the role of the P3 component lies in bridging stimulus perception and response execution^31,44^ in a dynamically evolving decision process, linking sensory encoding of stimulus perception to the preparation of action^42,43,45^, and/or response control^44^. In the current study, regression analysis showed that the latencies of the P3 peak amplitude, alongside PMP amplitudes, best predicted individual RTs on congruent trials per ethnicity. The PMP amplitudes alone predicted individual RTs on incongruent trials per ethnicity and were negatively correlated with the inhibition index. Differences in P3 latencies have been shown to correspond to the duration of stimulus evaluation^32,47^, with easier categorization reflected in larger amplitudes (e.g.^32,48^). This has been argued to be in line with the P3 component acting as an intermediate link on the dynamic formation of decision from stimulus encoding to motor preparation^42^. The results of the aforementioned regression analyses suggest that in a dynamic decision process, on congruent trials stimulus-response encoding happens early on (P3), linking perception of congruent stimulus-response matching to action for response initiation (PMP) (regardless of no differences in the P3 peak latencies across conditions; see supplementary material). This could reflect the facilitated encoding and motor execution of the perception-action link on congruent trials. On incongruent trials the encoding of the perceived SRC happens later on in the dynamic decision process, only at the time of the PMP component, potentially reflecting the enhanced difficulty of disengaging from the perceived task-irrelevant movement to prepare for, or potentially, initiate response execution. The role of the PMP, as mentioned above, to index motor initiation and its role in a decision formation process, by sharing overlap with a response-locked P3 component, may not exclude each other. Rather, it could be suggested that the integrative role of the response-locked P3 leading to motor preparation and the role of the PMP for motor initiation are overlapping processes and components. Future studies should aim to clarify the distinct and/or overlapping functions of the PMP and the response-locked P3 in the motor domain and whether the components reflect the same underlying neural processes. Considering that only on congruent trials, P3 latencies and PMP mean amplitudes together predicted individual mean RT’s, and P3 peak and PMP mean amplitudes correlated with each other (see supplementary material), the two components might overlap only on congruent trials in the present task. Furthermore, this could extend the investigation of combined stimulus- and response-locked P3 amplitudes to offer valuable insights into the link between perception and action as a dynamic decision process. The PMP may reflect motor-related self-other distinction in the initiation of movement, including, in a potential overlapping function with the response-locked P3 component, motor preparation. The present results thus allow new insights into the neural dynamics in motoric interference processing linking perception to action.

## Conclusion

This study provides new insights into the temporal processes underlying automatic imitation, and its modulation by ethnicity. Stimulus processing was influenced by both ethnicity and SRC presentation, while at the response execution stage only congruency influenced ERPs. Neural dynamics preceding response execution predicted the automatic imitation effect for White and, alongside a component of the SRC processing stage, for Black hands. Individual mean RTs on congruent trials were predicted by the P3 peak latency and PMP amplitudes, whereas PMP amplitudes alone predicted RTs on incongruent trials. Stimulus categorization of color/ethnicity may thus be reflected during stages of stimulus (i.e. color/ethnicity) processing, interacting with SRC processing at a later time point of this stage, but not being reflected in the overt behavioral response or the neural correlates immediately before and after movement execution. We suggest that our N190 results may index visual self-other distinction, concerned with color/ethnicity of the presented hands, whereas the PMP results may reflect motor-related self-other distinction regardless of ethnicity at movement initiation. The neural dynamics evolving in the stimulus-locked P3 and the PMP components, which may overlap with a response-locked P3, could reflect a dynamical decision process linking perception to action.

The absence of modulation of the behavioral response by ethnicity suggests that the EIIT, extending a well-established imitation-inhibition paradigm with stimuli of diverse color/ethnicity, may be used for participants of various ethnicities. This study can thus encourage the enhanced use of paradigms designed to fit various ethnicities and enhance generalizability in psychological research (for arguments regarding enhanced ethnic and socio-cultural equality in psychological research see e.g.^49–51^). We suggest using the EIIT, including both White and Black hand stimuli, to study automatic imitation. The presentation of ethnically diverse stimuli in cognitive (neuro)science could maximize generalizability of obtained findings in the field.

## Methods

### Participants

Thirty right-handed White participants took part in the EEG experiment and received a financial compensation of 25 Euro. One male participant was excluded due to incomplete EEG data, the final sample consisted of 29 individuals (21 women, *mean* age: 25.25 years, *SD* = 4.59 years). Participants were recruited via a local recruitment platform and social media. Participants’ handedness was assessed using the Edinburgh Handedness Inventory^52^. They reported no medical history of neurological, cardiovascular, or psychiatric disorders, and had normal or corrected to normal vision. The study was approved by the ethics committee of the University of Vienna and carried out in accordance with the Declaration of Helsinki (revision of 2013). As this was, to our knowledge, the first study investigating ERPs of automatic imitation focusing on their modulation by ethnicity, we took an explorative approach and recruited a middle-sized sample in comparison to related studies (e.g.^17,53^). Post-hoc power analyses suggested that the power of our exploratory approach was high for nearly all tested effects (1-β > 0.94).

### The ethnic imitation-inhibition task (EITT)

#### Stimuli and experimental design

Participants performed the ethnic imitation-inhibition task (EIIT), a modified version of the imitation-inhibition task by Brass *et al*.^3^ and the Social-Affective Mimicry Task (SAMT)^7,8^. The EIIT presents White and Black hands as task-irrelevant stimuli (wearing beige gloves, ethnicity visible at the wrist).

Automatic imitation paradigms rely on stimulus-response compatibility (SRC) effects. Task-relevant information is defined via task-instructions and mapped onto a number cue (‘1’ or ‘2’) displayed between index and middle finger of the task-irrelevant hand, triggering an index or middle finger lifting movement. Simultaneously, a task-irrelevant index or middle finger lifting movement is presented (see^3^). On stimulus-response *congruent* trials the task-relevant and task-irrelevant stimuli match; e.g. the task-relevant cue indexes a middle finger lifting movement, which is simultaneously depicted. This leads to *facilitation of motor execution* and faster *response execution*. On stimulus-response *incongruent* trials task-relevant and task-irrelevant stimuli do not match; e.g. the task-relevant cue indexes a middle finger lifting movement, while an index finger movement is depicted. This leads to *interference of motor execution* and slower *response execution* (see for example^1,3^). On baseline trials, the task-irrelevant hand does not perform any movements. Based on these three conditions, we calculated three indices: The difference in mean response times to incongruent and congruent trials is used as a measure for the automatic imitation effect (see also for example^3^). Moreover, the difference between mean response times of baseline and congruent, respectively incongruent and baseline trials was used to establish a baseline corrected response facilitation index (FI_c_) and inhibition index (II_c_) (see also literature on attentional bias, e.g.^54,55^).

The hand stimulus (frontal shot of a left hand) mirrored the right hand of the participant. Importantly, the hand was wearing a beige cotton glove and ethnicity was only detectable at the wrist of the hand. This was done to exclude confounding perceptual and attentional effects on task performance and modulation of mimicry caused by low-level color differences between conditions. Such confounding effects could have otherwise been caused by e.g. the visual contrast between the color of the fingers surrounding the black number cues on a grey square, or different perceptions and hence reactions to the movement of dark (i.e. Black, African-American) vs. bright (i.e. White, Caucasian) objects, respectively hands. Participants were also asked to wear the same beige glove throughout the experiment to ensure maximum comparability between the task-irrelevant hand on screen and participants’ hands. Potential effect of the modulation of automatic imitation would thus be due only to the subtle ethnicity manipulation. The target cue, the numbers “1” and “2”, were displayed in black font on a grey square and presented between index and middle finger of the task-irrelevant hand stimuli.

Moreover, in order to avoid perceptual differences or attentional effects driven by differential luminance characteristics of the ethnically diverse hand wrists, hand stimuli were matched for luminance using the SHINE (Spectrum, Histogram, and Intensity Normalization and Equalization) toolbox^56^ for Matlab 8.3 (TheMathworks, Inc., MA).

Stimuli were presented using two consecutive frames to simulate finger lifting movement (as opposed to the set-up of the SAMT used in^7,8^ using 4 frames) to minimise the influence of ERP component overlap induced by rapidly changing frames in the EEG/ERP signal (Fig. 1). The overall timing was nevertheless comparable between the SAMT and the EIIT, as was the presentation of the displayed hands in the lower half of the frame. The timing was set to a variable inter-trial interval with a random duration of 1200–1800 ms, showing a white fixation cross on a black screen between finger lifting movements. The first frame, presenting the hand stimuli without the number cue, was shown for 1534 ms; the second frame displayed the target cue and the respective task-irrelevant finger-lifting movement (respectively no movement in baseline trials) for another 1534 ms.

### Procedure

After signing the informed consent form, an EEG cap with 58 equidistantly mounted Ag/AgCl electrodes was applied (model M10, EASYCAP, GmbH, Herrsching, Germany). Two additional electrodes were applied one cm above and below the right eye to record vertical eye-movements (electrooculogram; EOG). Furthermore, four facial electromyographic electrodes were applied, but their data are not in the scope of the current study. A skin-scratching procedure ensured electrode impedances below 4 kΩ prior to recording^57^.

Participants were then seated in a dim-lighted, sound attenuated and electrically shielded chamber for EEG-recording. Seating distance was held constant at about 70 cm in front of a 19″ cathode ray tube (CRT) monitor (Sony GDM-F520; 85 Hz refresh rate). E-Prime 2.0 (Psychology Software Tools, Inc., Sharpsburg, PA) was used for stimulus presentation. Display resolution was set at 1280 × 1024 pixels (300 dpi). The experiment began by presenting detailed instructions, in which participants were asked to keep the keyboard number pad key “1” pressed with the index finger and the “2” key with the middle finger. Furthermore, instructions stated to lift the index finger whenever a “1”, and the middle finger whenever a “2” was presented on the screen. Participants were also instructed that any other stimuli presented on screen were not relevant to their task. In line with these instructions, potential modulation of the automatic imitation effect by the ethnically diverse hand stimuli occurred implicitly. The task consisted of 50 trials per condition (Ethnicity: White and Black, and Congruency: Congruent, Incongruent and Baseline) and target-cue (each 25 per finger, i.e. index and middle finger), resulting in a total of 300 trials, presented randomly. Practice trials (10 trials) were carried out before participants started the actual task. After 100 and 200 trials, short breaks were introduced. Total task duration was approximately 30 minutes.

EEG signals were collected with a DC-amplifier setup (NeuroPrax, neuroConn GmbH, Ilmenau, Germany) within a frequency range of DC to 500 Hz and sampled at 1000 Hz for digital storage.

### Post-experimental questionnaire

#### Attitudes towards Blacks scale

Explicit ethnic bias was measured with the Attitudes towards Blacks Scale^58^ after EEG measurements. The scale consists of 20 statements on attitudes towards Black people. Participants were asked to rate these statements on a seven-point scale ranging from “1”, indicating strong disagreement, to “7”, indicating strong agreement with the statement. Thus, a low mean score implies negative explicit attitudes, whereas a high score implies a favourable explicit attitude towards Black people.

### Data analysis

#### Analysis of behavioral data

Only data from correct trials entered data analysis. A winsorization procedure was applied to subjects’ individual mean response times (RT) per condition and target cue to account for outliers before further statistical analyses was conducted. Including outlier values in the analysis violates assumptions of general linear model estimations^59,61^. Therefore, as suggested by^60^, mean RTs higher than the 75^th^ percentile plus 1.5 times the interquartile range, and mean RTs lower than the 25^th^ percentile minus 1.5 times the interquartile range of the conditions and per target cue were replaced with the maximum or the minimum RT in the corresponding condition. The automatic imitation effect was calculated as the difference measure of participants´ mean RT on incongruent minus the mean RT on congruent trials (separate for White and Black stimuli). A dependent t-test was calculated to assess differences between the automatic imitation effect for White and Black Hands. Moreover, a 2 × 3 repeated measures ANOVA was conducted on the mean RT of participants with the within-subject factors of Ethnicity (White and Black) and Congruency (Congruent, Incongruent and Baseline). We furthermore calculated the baseline adjusted facilitation (FI_c_) and inhibition (II_c_) indices per ethnicity condition as the difference between mean response times of baseline and congruent (i.e. FI_c_), respectively incongruent and baseline trials (i.e. II_c_)^54,55^. We carried out dependent t-tests to compare the facilitation and inhibition indices per ethnicity condition. Baseline trials were included only in behavioural, but not in ERP analysis where the focus was on investigating neural processing of automatic imitation in incongruent and congruent trials.

Error rates in the EIIT were low, with participants’ mean error rates across congruent, incongruent and baseline trials of 1.42% (Mean = 4,41, SD = 4,021; Minimum: 1, Maximum: 17 errors). The error rate was thus too low in most participants to provide analyzable relevant information regarding the modulation of the automatic imitation effect and was not further included in the analysis.

#### EEG data

EEGLAB 13.3.2b^62^ implemented in Matlab 8.3 (The Mathworks, Inc., MA) was used for offline EEG data analysis. Data was down-sampled to 500 Hz, high (0.1 Hz) and low-pass filtered (30 Hz), and afterwards re-referenced to linked mastoids. Subsequently, extended infomax independent components analysis (ICA)^63,64^ was applied to detect eye movement-related artifacts. After discarding these artifacts, data segments of the possible conditions were extracted in three analysis steps: First, for the period of stimulus processing, stimulus-locked data analysis was carried out on the first frame of stimulus presentation with the factor Ethnicity (White and Black, i.e. ethnicity presentation) and on the second frame with the factors Ethnicity and Congruency (Congruent and Incongruent, i.e. SRC presentation) – as with behavioral data, only correct trials were analyzed. For this stimulus-locked data analysis, data segments were extracted 200 ms prior to stimulus onset until 1534 ms post stimulus onset per trial. The mean of the 200 ms interval prior to stimulus onset served as baseline interval. Second, response-locked data analysis was carried out with factors Ethnicity and Congruency for correct trials, which had been averaged time-locked to participants’ responses. For response-locked data analysis, data segments were extracted from −400 before to 600 ms after response execution for data analysis. The interval from −400 until −200 ms pre response served as baseline interval.

A semi-automatic artifact correction eliminated trials with voltage values exceeding ±75 µV or voltage drifts >50 µV. Marked trials (by the EEGLAB algorithms) were excluded if revision by visual inspection confirmed artifact affliction. Artifact-free segments were averaged per participant and condition first, for stimulus processing: Ethnicity presentation (frame 1, on average 117.45 trials, SD = 16.21): White vs. Black; SRC presentation (frame 2 stimulus-locked, on average 41.13 trials, SD = 4.45): White-congruent, White-incongruent, Black-congruent, Black-incongruent; Second for response execution (frame 2 response-locked, again on average 41.13 (SD = 4.45) artifact-free trials): White-congruent, White-incongruent, Black-congruent, Black-incongruent. In accordance to the behavioral analysis of the automatic imitation effect, we also calculated difference measures to address the automatic imitation effect as the difference of mean amplitudes of incongruent minus congruent trials per Ethnicity (White vs. Black) for second frame data. This was done to investigate which ERP components would predict the magnitude of the individual automatic imitation effect, as well as the RAP.

Time windows and electrode locations for ERP component quantification were determined by literature and visual data inspection since this was one of the first studies to investigate temporal dynamics of automatic imitation and its modulation by ethnically diverse hands. Please refer to Supplementary Materials for a detailed depiction of the current electrode layout and electrodes chosen for analyses.

First, we calculated overall global field power (GFP)^65–67^ to specify and/or validate a predefined timeframe of interest via assessing overall peak latency of the components of interest. Second, we inspected grand means and topographical maps of the previously defined time frames to define electrode clusters of interest.

To investigate the stage of stimulus processing during ethnicity presentation in the EIIT (stimulus-locked to frame 1), the N100 and the LPP were analyzed (please see supplementary material for additional analyses of posterior P2 peak amplitudes). N100 mean amplitudes were extracted 110–160 ms after stimulus onset within a cluster comprising right-hemispheric electrodes R27 and R30, and left-hemispheric electrodes L24 and L28. LPP mean amplitudes were extracted 400–800 ms after stimulus onset at an occipital cluster comprising the right-hemisphere electrodes R27 and R30, the left-hemispheric electrodes L24 and L28, and midline electrode Oz (L30). N1 and LPP amplitudes were analyzed with dependent t-tests (Ethnicity: White vs. Black).

Also in the stage of stimulus processing, during SRC presentation (stimulus-locked analysis of frame 2; presentation of task-relevant information concurring with task-irrelevant stimulus), we conducted peak value extraction for N190 and P3 components, since both ERPs show rather steep and distinct peaks. Amplitude courses were averaged participant- and condition-wise within the respective electrode clusters. N190 peaks were assessed as most negative peaks within a time window of 150–240 ms after frame 2 onset in an occipital left and right hemispheric clusters comprising electrodes R22, R27, R30 (right) and L20, L24, L28 (left), P3 peaks were analyzed as most positive peaks in a parietal cluster comprising electrodes R25, R26, R29, R30, L22, Pz (L26), L27, L28, and Oz (L30) within a time window of 250 and 460 ms after frame 2 onset. The reported time windows for peak extraction were chosen to acknowledge inter-individual variation in peak latencies. P3 peak latencies were measured from frame 2 onset to the corresponding maximum in the respective time window. Of note, for the N190, we added the factor HEMISPHERE (Left vs. Right), according to Deschrijver *et al*.^12^.

Third, to investigate components linked to response execution (response-locked analysis of frame 2), we analyzed mean amplitudes of the PMP during 100 ms before to the actual response in a parietal cluster at electrodes R19, CPz (R24), R25, R29, L22, Pz (L26), and L27. The RAP was analyzed from 126 ms to 176 ms after response execution in a central cluster at electrodes FCz (R14), Cz (L16), CPz (R24), R15, R16, R19, R25, L12, and L17.Two-way repeated measures ANOVAs with the within-subject factors Ethnicity and Congruency were calculated separately for these ERPs.

We performed Pearson correlation per ethnicity and congruency condition of components showing a significant congruency effect, thus, the peak P3, as well as mean PMP and RAP amplitudes, with the corresponding facilitation (i.e. FI_c_) and inhibition (i.e. II_c_) index per ethnicity. This allowed to investigate whether facilitation or inhibition indices (baseline corrected) corresponded with stimulus congruency presentation in the stage of stimulus processing or movement-related potentials of components showing a significant Congruency effect (see supplementary material for additional correlation analysis). Moreover, we performed multiple regression models (stepwise method: forward). We entered ERPs, showing significant results, in the stage of stimulus processing, during ethnicity presentation, as well as the ERP congruency difference measures (i.e. mean amplitude difference of incongruent minus congruent trials) of components during congruency presentation and in the stage of response execution as independent variables (per ethnicity). As dependent variables, we entered (in separate models) the corresponding magnitude of the individual automatic imitation effect and the RAP difference measure (per ethnicity). This allowed us to investigate which ERP components predicted the magnitude of the individual automatic imitation effect and RAP amplitudes. Furthermore, in an exploratory approach, we also performed multiple regression models with all ERP amplitudes and peak latencies (i.e. P3) of significant results as independent and individual mean RTs per condition as dependent variables (separate model per condition including condition-specific ERP amplitudes and latencies as well as individual mean RTs).

Effect sizes of significant results are specified with partial eta squared (ηp2)^68^ and Cohen’s d; the alpha-level was set at p < 0.05 in both behavioral and EEG data analyses. Data availability statement: URLs/accession numbers/DOIs will be available only after acceptance of the manuscript for publication.

## Electronic supplementary material

Supplementary Information
