# Supplementary material for: Event-related potentials of automatic imitation are modulated by ethnicity during stimulus processing, but not during motor execution
Source: Sci Rep. 2018 Aug 24;8:12760. doi: 10.1038/s41598-018-30926-4 (PMC6109053; doi:10.1038/s41598-018-30926-4)
Supplement: Supplementary file 1 — Supplementary Information [file 41598_2018_30926_MOESM1_ESM.doc]

**SUPPLEMENTARY MATERIALS**

**Event-related potentials of automatic imitation are modulated by ethnicity during stimulus processing, but not during motor execution**

Birgit Rauchbauer & Daniela M. Pfabigan, Claus Lamm

1. **Additional Methods Section**
   1. Electrode layout

The following figures display the electrode montage used in the current study according to the international 10-10 system implemented by Easycap GmbH (Herrsching, Germany) in their model M10. Coloured marks denote the respective electrode clusters per ERP. Electrodes on the right hemisphere (R) are depicted on white background, electrodes on the left hemisphere (L) on grey background. Please note, electrode positions R10, L7, and L31 were not recorded in the current experiment.

**Figure S1**. Electrode clusters for ERPs during presentation of stimulus ethnicity (frame 1)


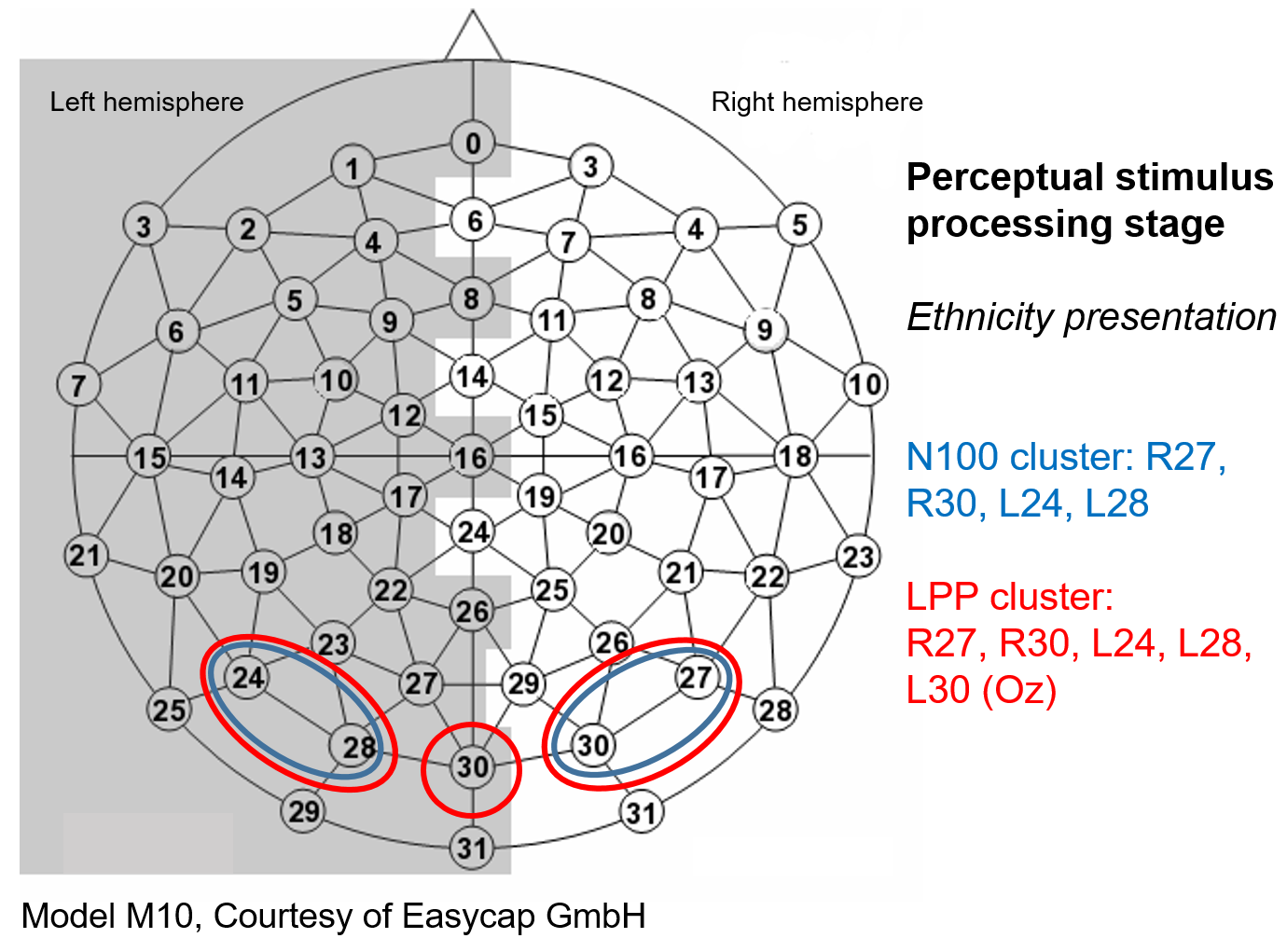


**Figure S2**. Electrode clusters for ERPs during stimulus-response compatibility (SRC) presentation (frame 2)


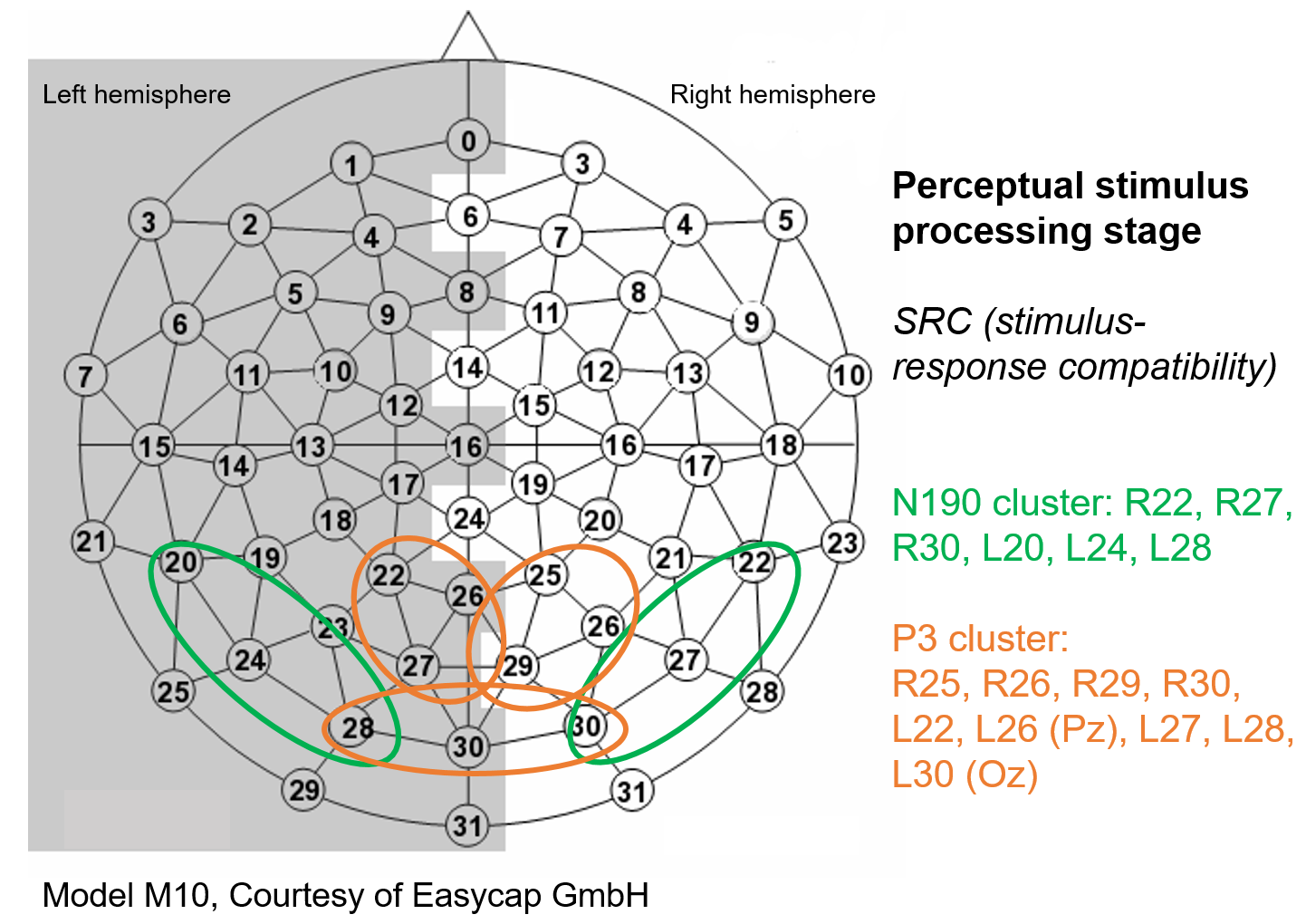


**Figure S3**. Electrode clusters for ERPs during response execution


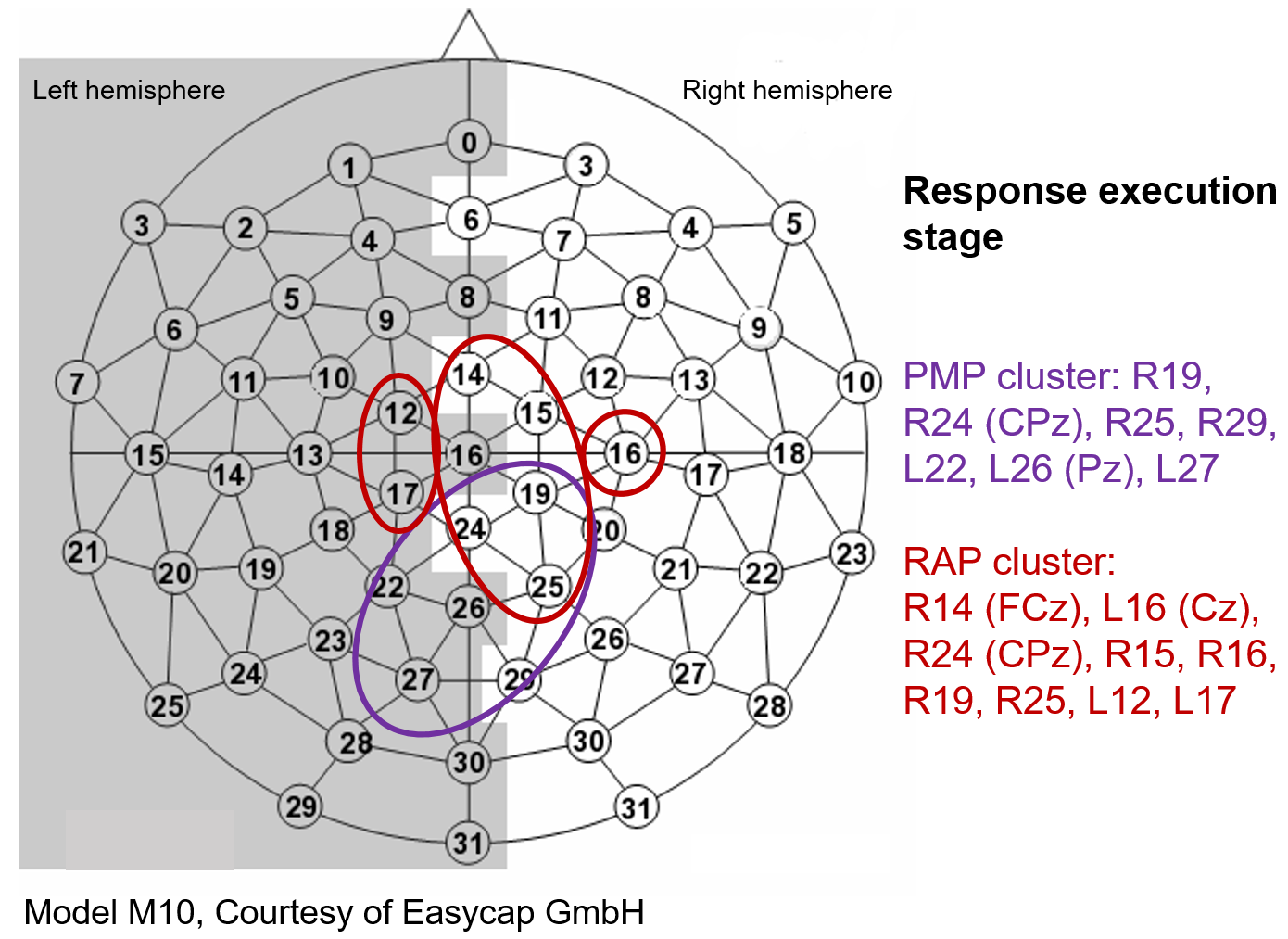


1.2 Data analysis during ethnicity presentation (stage of perceptual stimulus processing)

We assessed peaks of the distinct positive ERP deflection around 220 ms (P2 component) after the onset of frame 1 at the occipital cluster also used for LPP analysis (see Figure S1 for electrode locations and Figure 2 in the main document for amplitude courses). In detail, the most positive peaks in a time window of 150 – 350 ms after frame 1 onset were extracted per participant and condition (Black, White) in the occipital cluster.

1.3 Data analysis during stimulus-response compatibility presentation (stage of perceptual stimulus processing)

In addition to the peak analysis, we also analyzed N190 and P3 components with a mean amplitudes approach. We assessed mean amplitudes of the two ERPs at the same electrode clusters as used for peak amplitude analyses. The N190 was analyzed in the timeframe 170 – 220 ms after stimulus onset (see also (Deschrijver, Wiersema, & Brass, 2017), after confirming a peak at 190 ms with GFP). According to the topographical maps we used occipital left and right hemispheric clusters for the N190, comprising electrodes R22, R27, R30 (right) and L20, L24, L28 (left). The P3 component was analyzed in the timeframe 300 – 400 ms after stimulus-onset at a parietal cluster comprising electrodes R25, R26, R29, R30, L22, Pz (L26), L27, L28, and Oz (L30). Two-way repeated measures ANOVA with the within-subject factors Ethnicity and Congruency (Congruent vs. Incongruent) were calculated separately for the N190 and the P3. Of note, for the N190, we added the factor HEMISPHERE (Left vs. Right), according to Deschrijver et al., (2017).

In addition, we report variation in P3 peak latencies here, which was investigated with the same two-way repeated measures ANOVA design (Ethnicity x Congruency).

1.4 Additional correlation analyses

Also, we carried out Pearson correlation between the P3 peak amplitudes’ latencies and participants’ mean RTs on corresponding congruent and incongruent trials to investigate the P3 latencies’ influence on RTs. Furthermore, to investigate the relationship between the P3 and the PMP amplitudes, we performed Pearson correlation between these two components. This was done to test whether P3 amplitude variation influenced PMP amplitudes, and, moreover, whether the P3 and the PMP components actually reflected an overlapping ERP component considering also their shared topographical distribution on the scalp.

1. **Additional Results Section**
   1. P2

A paired t-test revealed a significant difference in P2 peak amplitudes between ethnicity, with more positive-going P2 amplitudes for Black (9.85µV, SD=3.64) than White (9.18µV, SD=3.57) hand trials (t(28) = 3.368, p = .002, d=0.19).

2.2 N190 mean amplitudes

The 2 x 2 x 2 repeated measures ANOVA with the factors Ethnicity, Congruency, and Hemisphere revealed a trend result for the factor Congruency in the expected direction of more negative N190 amplitudes for incongruent trials (F(1, 28) = 3.127, p = .088, partial η2 = .100; Congruent: M = .036, SE = .336; Incongruent: M = -.193, SE = .319). The factor Ethnicity was significant, with more negative N190 amplitudes for Black than White hands (F(1, 28) = 9.795, p = .004, partial η2 = .259; White: M = .125, SE = .329; Black: M = -.282, SE = .327). Furthermore, an interaction effect of Ethnicity x Hemisphere was observed (F(1, 28) = 5.732, p = .024, partial η2 = .170). Planned pairwise comparisons (Bonferroni corrected p ≤ .0125) revealed a significant difference in N190 mean amplitudes on the left hemisphere for Black compared to White hand stimuli (t(28) = -3.666, p = 0.001; left Black hands: M = -.444, SE = .390, left White hands: M = .163, SE = .435). No further significant differences were found (all F-values < 2.54, all p-values ≥ .122).

2.3 P3 mean amplitudes

The 2 x 2 repeated measures ANOVA with the factors Ethnicity and Congruency showed a significant main effect of Ethnicity with more pronounced P3 amplitudes for White than Black ethnic stimuli (F(1, 28) = 5.73, p < .024, partial η2 = .17; Black: M = 8.43, SE = .61; White: M = 8.79, SE = .57). Also the main effect of Congruency was significant with more positive P3 amplitudes for congruent than incongruent trials (F(1, 28) = 21.60, p < .001, partial η2 = .43; Congruent: M = 9.07, SE = .60; Incongruent: M = 8.15, SE = .58). Their interaction was not significant (F(1, 28) = .906, p = .349).

The following tables report means and SD of behavioral and ERP data.

Please note: Calculation of the reported indices is described in the main document.

Please note: R = right hemispheric electrodes; L = left hemispheric electrodes; ERP amplitude variation was assessed as mean amplitudes apart for the lines N190 R peaks, N190 L peaks, and P3 peaks.

2.4 P3 peak latency analysis

The 2 x 2 repeated measures ANOVA with the factors Ethnicity and Congruency showed no significant effect of Ethnicity (F(1, 28) = .76, p = .390, partial η2 = .03) or Congruency (F(1, 28) = 1.48, p = .234, partial η2 = .05), or their interaction (F(1, 28) = .02, p = .895, partial η2 = .001; Black Congruent: M = 369,38, SD = 25.74; Black Incongruent: M = 363.72, SD = 29.46; White Congruent: M = 365.38, SD = 35.48; White Incongruent: M = 360.76, SD = 35.17). These findings suggests that the processing speed did not differ for the four experimental conditions.

2.5 Additional correlation analyses

Participants’ mean RTs on White and Black congruent, but not incongruent, trials were significantly positively correlated with P3 peak latencies (Congruent trials: Black: r = .405, p = .029; White: r = .485, p = .008; Incongruent trials: all p-values ≥ .153). Furthermore, we correlated P3 peak and PMP mean amplitudes to investigate, whether the two components were related to each other. Results showed P3 peak and PMP mean amplitudes to be significantly positively correlated for both White and Black congruent, but not incongruent trials (Congruent trials: Black: r = .563, p = .001; White: r = .587, p = .001; Incongruent trials: all p-values ≥ .106).

2.6 Additional figures depicting amplitude courses of baseline trials

Please refer to the following figures of frame 2 – stimulus- and response-locked – depicting also amplitude courses of the baseline trials [base] in addition to congruent and incongruent trials per ethnicity.

**Figure S4. Grand average of stimulus-locked P3 component**

**
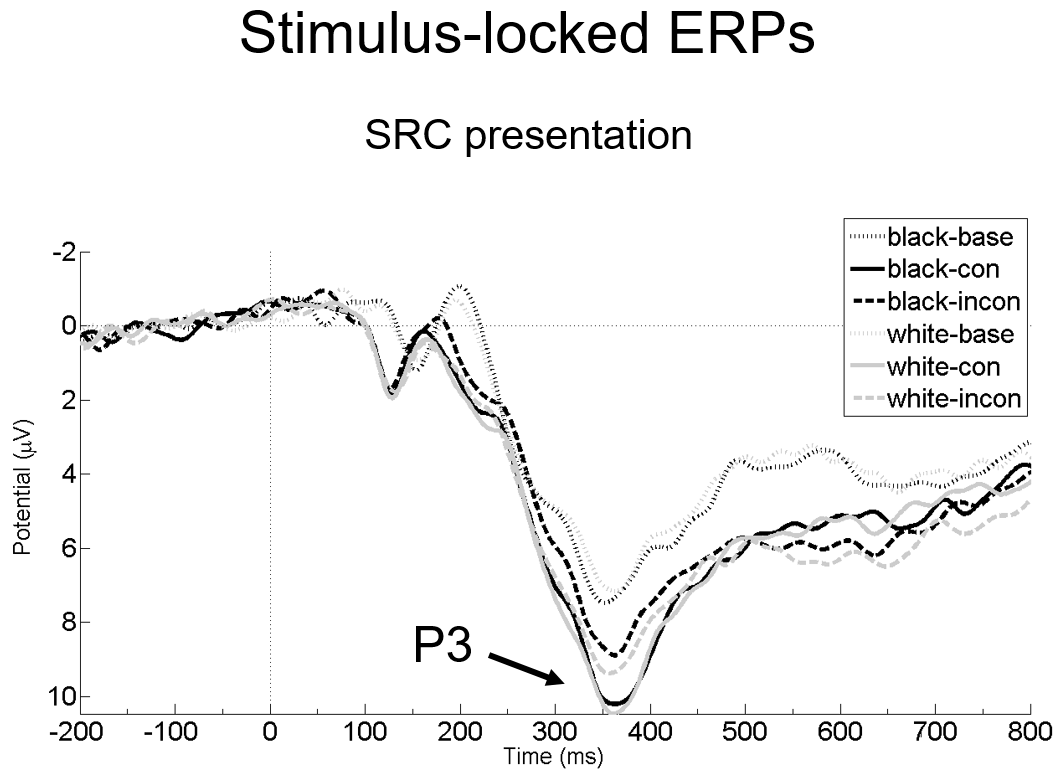
**

**Figure S4.** Grand average of stimulus-locked P3 component. Mean P3 amplitudes after onset of Frame 2 at merged electrodes R25, R26, R29, R30, L22, Pz (L26), L27, L28, and Cz (L30) per Congruency (baseline [base] vs. congruent [con] vs. incongruent [incon]) and Ethnicity (black vs. white). Negative amplitudes are drawn upwards by convention. Stimulus presentation started at 0, indicated by a ticked vertical line. For demonstrational purposes, only the first 800 ms of the frames are depicted.

**Figure S5. Grand average of stimulus-locked N190 component**


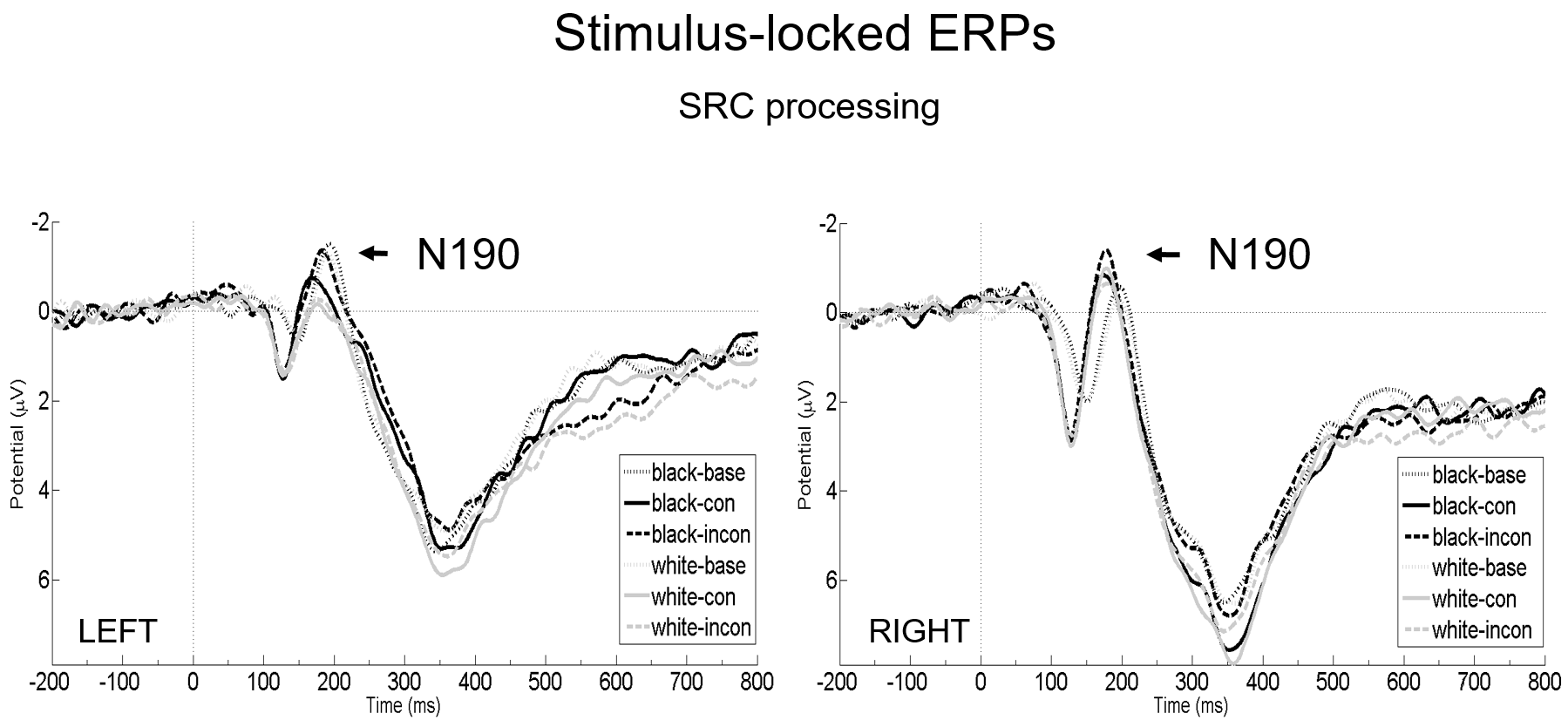


**Figure S5.** Grand average of stimulus-locked N190 component. Left panel depicts left hemispheric, right panel depicts right hemispheric N190 amplitudes after onset of Frame 2 at merged electrodes R22, R27, R30 (right hemisphere) and L20, L24, L28 (left hemisphere) per Ethnicity (black vs. white) and Congruency (baseline [base] vs. congruent [con] vs. incongruent [incon]). Negative amplitudes are drawn upwards by convention. Stimulus presentation started at 0, indicated by a ticked vertical line. For demonstrational purposes, only the first 800 ms of the frames are depicted.

**Figure S6. Grand average of response-locked ERPs (PMP and RAP)**


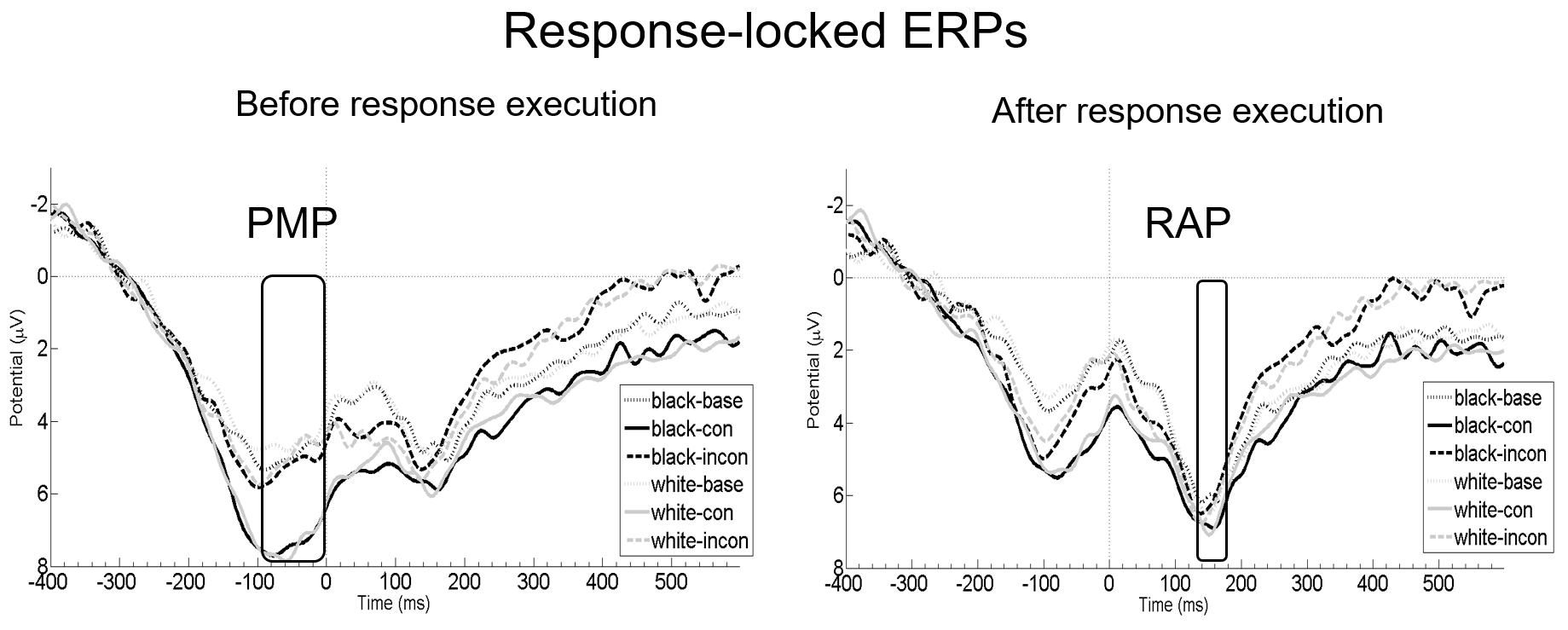


**Figure S6.** Grand average of response-locked ERPs (PMP and RAP). Left panel: Mean PMP amplitudes 100 ms prior until the button press (rectangle) at merged electrodes R19, CPz (R24), R25, R29, L22, Pz (L26), and L27, per Congruency (baseline [base] vs. congruent [con]vs. incongruent [incon]) and Ethnicity (black vs. white). Right panel: Mean RAP amplitudes 126 – 176 ms after response execution (rectangle) at merged electrodes CFz (R14), Cz (L16), CPz (R24), R15, R16, R19, R25, as well as L12 and L17 per Congruency (baseline [base] vs. congruent [con] vs. incongruent [incon]) and Ethnicity (black vs. white). Negative amplitudes are drawn upwards by convention. Response execution started at 0, indicated by a ticked vertical line.

1. **Additional Discussion**

3.1 P2 component

The P2 showed enhanced peak amplitudes in response to Black as compared to White hand trials. This, and the topographical distribution of the P2 being occipital rather than fronto-central (see for example Potts, 2004) suggests that the P2 reflects enhanced visual attention processes to Black as compared to White stimuli in the current study. This is in line with research on ethnicity effects, showing an enhanced P2 component in response to Black as compared to White faces, due to enhanced attention and vigilance in response to salient Black faces when seen by White participants (Ito & Bartholow, 2009; Ito & Senholzi, 2013; Ito & Urland, 2003, 2005). The effect has been shown reversed in Black participants, showing enhanced amplitudes for White rather than Black faces, which suggests the P2 component to reflect a social categorization of group membership rather than color differences alone (Dickter & Bartholow, 2007). Future studies may aim to investigate the differences in neural dynamics in the EITT as a function of ethnicities; including both White and Black participants.

3.2 N190 and P3 mean amplitudes

We report N190 and P3 mean amplitudes in this document to give a comprehensive overview regarding all conducted analyses.

The overall pattern of results was comparable for N190 peak and mean amplitudes analysis. A distinct main effect of Ethnicity (Black hands > White hands) was observed applying both analysis methods. Please refer to the main document for discussion.

Regarding P3 amplitudes, results differed slightly for the two quantification methods. While the peak assessment revealed a significant interaction of Ethnicity and Congruency, the mean amplitude assessment showed two significant main effects, but no interaction. This discrepancy was driven by a larger difference between congruent and incongruent White hand trials assessed with the mean (t(28)=3.535, p=0.001) than the peak amplitudes approach (t(28)=2.051, p=0.050). Table S2 provides means and standard deviation (SD) for both approaches. It can be seen that standard deviations were larger for the peak than the mean amplitudes method, which could be one explanation for the slightly diverging results. Nevertheless, we chose to focus our interpretation in the main document on the results of the P3 peak amplitudes approach since it takes individual component latency into consideration. Although mean amplitude approaches are less susceptible to noise in the EEG data, they show less sensitivity regarding inter-individual differences in peak latency since the same analysis time window is applied to all participants (Luck, 2005).

3.3 Additional discussion on the LPP component

Another important aspect we have to address is the notion that any SCR paradigm could also be framed as a simple variant of a classical S1-S2 paradigm. Concerning the current study, frame 1 could also be regarded as S1 stimulus, inducing expectations about the upcoming frame 2, which could be regarded as S2 stimulus. In light of this categorization, one could interpret the slow amplitude changes starting 300 ms after frame 1 onset also as variation of the contingent negative variation (CNV) component (Falkenstein, Hoormann, Hohnsbein, & Kleinsorge, 2003; Gómez, Flores, & Ledesma, 2007). Negative potential shifts are reported to reflect anticipatory attention and preparation of effortful processes. In the current study, we assessed slow potential changes in the time window 400-800 ms after frame 1 onset at a parieto-occipital electrode cluster (based on global field power and topographical results) and interpreted these amplitude changes in light of the LPP component. However, if we take over the viewpoint that SCR paradigms are a simple variant of S1-S2 paradigms, one could also interpret the observed amplitude changes as CNV component. Based on previous CNV literature, our investigated time interval should reflect early parts of the CNV component, which are considered reflecting an orienting ERP with a frontal minimum (Gaillard, 1976). More recent theoretical accounts link (early) CNV amplitude variation also to temporal preparation (Ng, Tobin, & Penney, 2011) as well as to temporal production or temporal reproduction tasks (Kononowicz & Van Rijn, 2011; Macar & Vidal, 2003). Thus, our results showed that White hand stimuli led to more negative amplitude shifts than Black hand trials. This could be interpreted as heightened early orienting response as well as preparatory efforts in the time domain (regarding when an upcoming motor response had to be performed, which had a fixed duration in the current paradigm) for stimuli of the same ethnicity as the participants. However, the topographical distribution of the observed amplitude variation at posterior electrodes is not in line with the mentioned CNV studies, which rather observed frontal minima for the early CNV component. Consequently, this observation limits the possibility that the current experimental task set-up is a reliable S1-S2 paradigm version, which could be applied to investigate CNV amplitude variation. Therefore, we maintain interpreting the slow amplitude changes in frame 1 as LPP component in the main document.

**References**

Deschrijver, E., Wiersema, J. R., & Brass, M. (2017). The influence of action observation on action execution: Dissociating the contribution of action on perception, perception on action, and resolving conflict. *Cognitive, Affective, & Behavioral Neuroscience*, *17*(2), 381–393.

Dickter, C. L., & Bartholow, B. D. (2007). Racial ingroup and outgroup attention biases revealed by event-related brain potentials. *Social Cognitive and Affective Neuroscience*, *2*(3), 189–198.

Falkenstein, M., Hoormann, J., Hohnsbein, J., & Kleinsorge, T. (2003). Short‐term mobilization of processing resources is revealed in the event‐related potential. *Psychophysiology*, *40*(6), 914–923.

Gaillard, A. W. K. (1976). Effects of warning-signal modality on the contingent negative variation (CNV). *Biological Psychology*, *4*(2), 139–153.

Gómez, C. M., Flores, A., & Ledesma, A. (2007). Fronto-parietal networks activation during the contingent negative variation period. *Brain Research Bulletin*, *73*(1–3), 40–47.

Ito, T. A., & Bartholow, B. D. (2009). The neural correlates of race. *Trends in Cognitive Sciences*, *13*(12), 524–531.

Ito, T. A., & Senholzi, K. B. (2013). Us versus them: Understanding the process of race perception with event-related brain potentials. *Visual Cognition*, *21*(9–10), 1096–1120.

Ito, T. A., & Urland, G. R. (2003). Race and gender on the brain: electrocortical measures of attention to the race and gender of multiply categorizable individuals. *Journal of Personality and Social Psychology*, *85*(4), 616.

Ito, T. A., & Urland, G. R. (2005). The influence of processing objectives on the perception of faces: An ERP study of race and gender perception. *Cognitive, Affective, & Behavioral Neuroscience*, *5*(1), 21–36.

Kononowicz, T. W., & Van Rijn, H. (2011). Slow potentials in time estimation: the role of temporal accumulation and habituation. *Frontiers in Integrative Neuroscience*, *5*, 48.

Luck, S. J. (2005). *An introduction to the event-related potential technique*. Cambridge, Massachsetts: The MIT Press.

Macar, F., & Vidal, F. (2003). The CNV peak: an index of decision making and temporal memory. *Psychophysiology*, *40*(6), 950–954.

Ng, K. K., Tobin, S., & Penney, T. B. (2011). Temporal accumulation and decision processes in the duration bisection task revealed by contingent negative variation. *Frontiers in Integrative Neuroscience*, *5*, 77.

Potts, G. F. (2004). An ERP index of task relevance evaluation of visual stimuli. *Brain and Cognition*, *56*(1), 5–13.
